# DNA methylation patterns of transcription factor binding regions characterize their functional and evolutionary contexts

**DOI:** 10.1186/s13059-024-03218-6

**Published:** 2024-06-06

**Authors:** Martina Rimoldi, Ning Wang, Jilin Zhang, Diego Villar, Duncan T. Odom, Jussi Taipale, Paul Flicek, Maša Roller

**Affiliations:** 1 European Molecular Biology Laboratory, European Bioinformatics Institute, Wellcome Genome Campus, Hinxton, Cambridge, CB10 1SD UK; 2 Department of Medical Biochemistry and Biophysics, Division of Functional Genomics and Systems Biology, Karolinska Institutet, Stockholm, SE 141 83 Sweden; 3 Cancer Research UK Cambridge Institute, University of Cambridge, Robinson Way, Cambridge, 0RE, CB2 UK; 4 Present Address Blizard Institute, Barts and The London School of Medicine and Dentistry, Queen Mary University of London, London, E1 2AT UK; 5 Present address Division of Regulatory Genomics and Cancer Evolution, German Cancer Research Center (DKFZ), Im Neuenheimer Feld 280, Heidelberg, 69120 Germany; 6 Applied Tumor Genomics Research Program, Research Programs Unit, Faculty of Medicine, University of Helsinki, Helsinki, Finland; 7 Department of Biochemistry, University of Cambridge, Cambridge, CB2 1GA UK; 8 Wellcome Sanger Institute, Wellcome Genome Campus, Hinxton, Cambridge, CB10 1SD UK; 9 Department of Genetics, University of Cambridge, Cambridge, CB2 3EH UK

**Keywords:** DNA methylation, Transcription factor binding, Evolution, Mammals

## Abstract

**Background:**

DNA methylation is an important epigenetic modification which has numerous roles in modulating genome function. Its levels are spatially correlated across the genome, typically high in repressed regions but low in transcription factor (TF) binding sites and active regulatory regions. However, the mechanisms establishing genome-wide and TF binding site methylation patterns are still unclear.

**Results:**

Here we use a comparative approach to investigate the association of DNA methylation to TF binding evolution in mammals. Specifically, we experimentally profile DNA methylation and combine this with published occupancy profiles of five distinct TFs (CTCF, CEBPA, HNF4A, ONECUT1, FOXA1) in the liver of five mammalian species (human, macaque, mouse, rat, dog). TF binding sites are lowly methylated, but they often also have intermediate methylation levels. Furthermore, biding sites are influenced by the methylation status of CpGs in their wider binding regions even when CpGs are absent from the core binding motif. Employing a classification and clustering approach, we extract distinct and species-conserved patterns of DNA methylation levels at TF binding regions. CEBPA, HNF4A, ONECUT1, and FOXA1 share the same methylation patterns, while CTCF's differ. These patterns characterize alternative functions and chromatin landscapes of TF-bound regions. Leveraging our phylogenetic framework, we find DNA methylation gain upon evolutionary loss of TF occupancy, indicating coordinated evolution. Furthermore, each methylation pattern has its own evolutionary trajectory reflecting its genomic contexts.

**Conclusions:**

Our epigenomic analyses indicate a role for DNA methylation in TF binding changes across species including that specific DNA methylation profiles characterize TF binding and are associated with their regulatory activity, chromatin contexts, and evolutionary trajectories.

**Supplementary Information:**

The online version contains supplementary material available at 10.1186/s13059-024-03218-6.

## Background

Gene regulation is a complex process that controls gene expression across cell types and time points. Key players in establishing tissue-specific expression are transcription factors, which bind to specific DNA sequences, and covalent modifications to the DNA such as DNA methylation (DNAm). Regulatory evolution has widely been studied in comparative analysis of transcription factor binding, but complementary studies of the evolution of DNAm are lacking.

There are several known mechanisms influencing transcription factor binding evolution. Transcription factor (TF) binding evolves rapidly: in mammals, it is characterized by frequent gain and loss of binding events even across short evolutionary time [1–5]. One mechanism of lineage-specific TF binding divergence is through transposable elements, which have repeatedly introduced novel binding sites in multiple lineages [6–9]. Another mechanism is sequence divergence, which can partly explain binding divergence. For example, a comparative study of a handful of liver-specific transcription factors in five mammals reported that more than 60% of binding losses could be explained by binding motif disruption through mutations or indels [10]. However, in the remaining 20–40% of lost binding events, the motif was unchanged. Furthermore, TF binding in the liver of rat and five mouse strains showed similar mutational rates between binding-conserved and binding-lost TF motifs [11], indicating that sequence divergence alone cannot explain TF turnover.

Despite the rapid rearrangements of the TF binding network [3, 12], gene expression of orthologous genes tends to be conserved in mammals [13, 14], likely due to the plasticity of the regulatory network [15]. For example, compensatory binding turnover in the proximity of lost events preserves regulatory network connectivity [10, 15] and the complexity of regulatory landscapes [16]. Finally, cooperative binding of multiple TFs [11] and clustered binding of a single TF [17] are more evolutionarily stable than lone binding events. Less is known about how epigenetic modifications of DNA evolve and affect the evolutionary dynamics of transcription factor binding.

DNA methylation (DNAm) is a chemical modification of DNA, most commonly the addition of a methyl group to the fifth position of cytosines (5-methylcytosine (5mC)), for those cytosines followed by a guanine (CpGs). The presence of CpG methylation can be measured in bulk tissues and cell types as a continuous frequency value comprised between 0 and 100% (or 0 to 1) through whole-genome bisulfite sequencing assays (WGBS) [18]. Most CpGs in mammalian genomes measure 0–10% and 70–100% methylation, indicating overall unmethylated and methylated nucleotides, respectively [19]. However, about one in 10 CpGs have intermediate levels, i.e. between 10 and 70% methylation [20], reflecting either the cell-to-cell variability of the bulk samples or epigenomic and transcriptional heterogeneity [21–23].

DNAm is largely recognized as a repressive epigenetic mark that often displays spatially correlated patterns across the genome [24]. Most transposable elements are repressed by 5mC modifications throughout their length [6, 25]. On the other hand, active regulatory regions are typically unmethylated, specifically CpG islands and active promoters and enhancers [26–28]. Genomic regions with intermediate methylation (IM) levels were shown to be widespread and conserved across species [29]. They typically co-localize with distal regulatory elements and can be reshaped upon transcription factor binding [20]. In fact, DNAm levels are tightly linked to functional and chromatin contexts. Transcriptional activity, TF binding, and chromatin remodelers have an impact on passive and active enzymatic processes that ultimately determine local patterns of methylation across the genome [30]. As a consequence, DNAm levels are highly predictive of regulatory activity [31–34].

DNA methylation was traditionally thought to inhibit transcription factor binding by physically preventing proteins from binding their target DNA sequences [35]. However, mounting evidence from in vivo and in vitro experiments now challenges this view. High-throughput in vitro assays such as protein microarrays and methyl-SELEX (systematic evolution of ligands by exponential enrichment) showed that not only TFs bind methylated motifs, but also that their binding affinity can be enhanced by 5mC [36–38]. Evidence that the methylation landscape can be remodeled in vivo by the binding of specific transcription factors such as the CCCTC-binding factor (CTCF) or the RE1 Silencing Transcription Factor (REST) has further challenged the traditional view [20]. Despite the experimental in vivo identification of a handful of TFs with modified specificity for methylated motifs [38, 39], the impact of cytosine methylation on the regulation of TF binding in distinct genomic contexts remains unclear.

We designed a comparative epigenomic study of DNA methylation patterns within TF binding regions. We generated whole-genome bisulfite-sequencing data from livers of five mammalian species (human, macaque, mouse, rat, and dog) and retrieved publicly available ChIP-sequencing data from five transcription factors in matched tissues. Four of the assayed transcription factors represent key components of the liver-specific regulatory network [40], namely CCAAT/enhancer-binding protein alpha (CEBPA), hepatocyte nuclear factor 4 alpha (HNF4A), One Cut Homeobox 1 (ONECUT1, also known as HNF6), and forkhead box protein A1 (FOXA1, also known as HNF3A). The final TF included in this study, CTCF, is a ubiquitous and multifunctional protein [41]. We used these datasets to characterize DNA methylation at TF binding regions, find different DNAm patterns within distinct functional genomic elements, and show that DNA methylation and TF binding co-evolve.

## Results

### Experimentally profiling DNA methylation in transcription factor binding regions

**Fig. 1:**
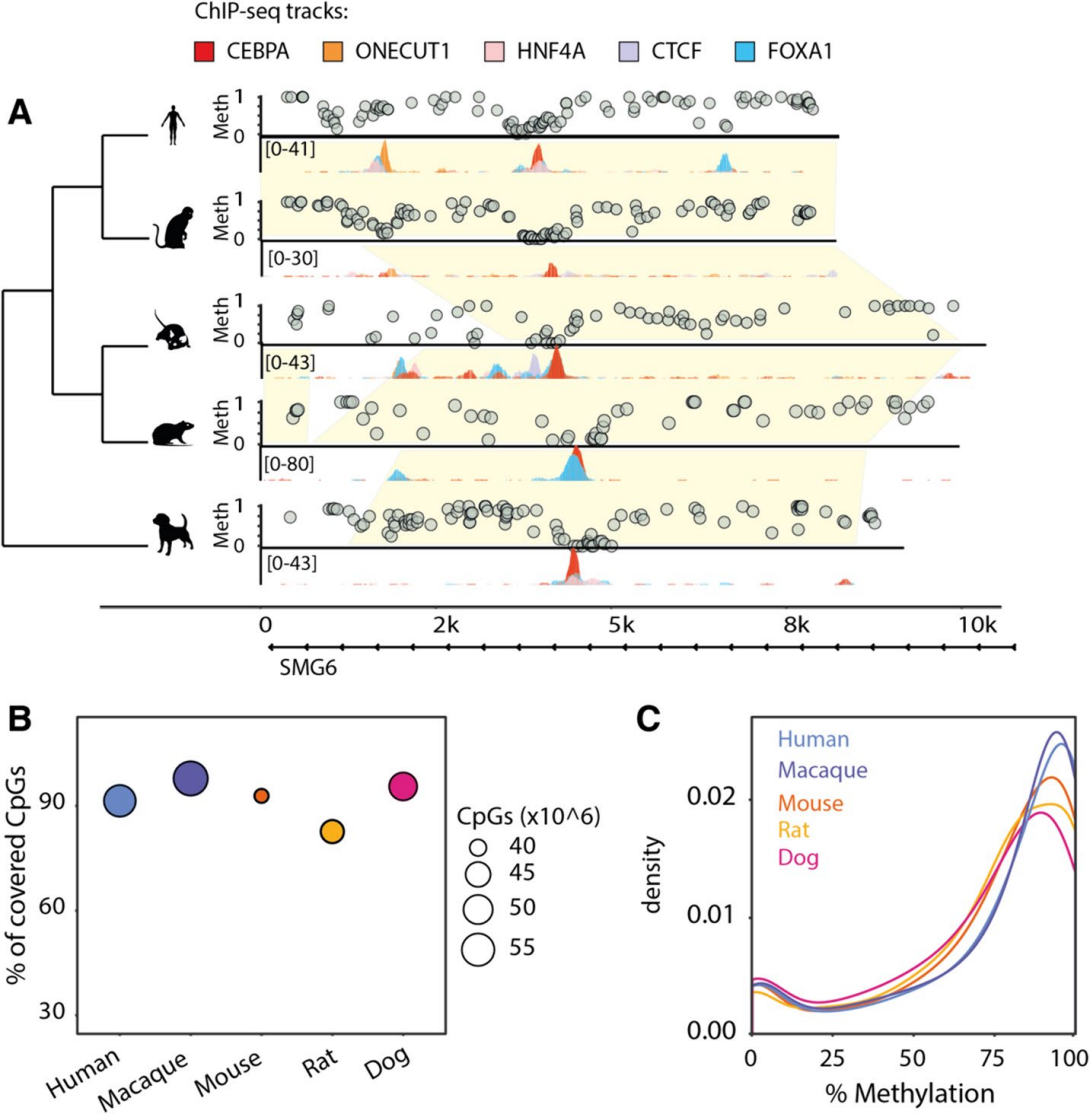
Experimentally mapping methylomes across mammals. **A** Example region: in vivo 5mC methylation and transcription factor binding around the SMG6 locus in livers isolated from five mammalian species. Yellow shades indicate EPO eutherian mammal alignment blocks (Ensembl version 98). For each species, levels of CpG methylation assayed with bisulfite sequencing are shown above the region, and binding of five transcription factors tracks (CEBPA, CTCF, FOXA1, HNF4A, ONECUT1) assayed through ChIP-sequencing are shown below. **B** Genomic coverage of WGBS data in each species. The *y*-axis shows the percentage, while the radius of each point denotes the total number of CpGs covered on the forward and reverse strands. **C** Genome-wide CpG methylation density distributions for each species. All distributions are bimodal, with the vast majority of CpGs hypermethylated

We obtained flash-frozen liver samples from five mammalian species (*Homo sapiens*, *Macaca mulatta*, *Mus musculus*, *Rattus norvegicus*, and *Canis familiaris*) and performed whole-genome bisulfite sequencing to assay genome-wide CpG methylation. We combined these results with previously published chromatin immunoprecipitation followed by high-throughput sequencing (ChIP-seq) data for tissue-specific and ubiquitous transcription factors. Specifically, we reanalyzed ChIP-seq data for CTCF, CEBPA, and HNF4A, in all five species; FOXA1 in all species but macaque; and ONECUT1 in all species but dog. This allowed us to determine the CpG methylation patterns at transcription factor binding regions and compare their evolutionary conservation across mammals (Fig. 1A). We profiled the methylation of 39–57 million CpGs in each species at an average of 6–15 × coverage (Supplementary Table 1), which accounted for 82–97% of all genomic CpGs (Fig. 1B). As previously reported [35], the distribution of genomic CpG methylation is bimodal, with the highest between 80 and 100% methylation and the lowest between 0 and 10% (Fig. 1C). These coordinated datasets enabled us to investigate relationships between DNA methylation and transcription factor binding.

### Transcription factors can bind DNA of all methylation levels

**Fig. 2:**
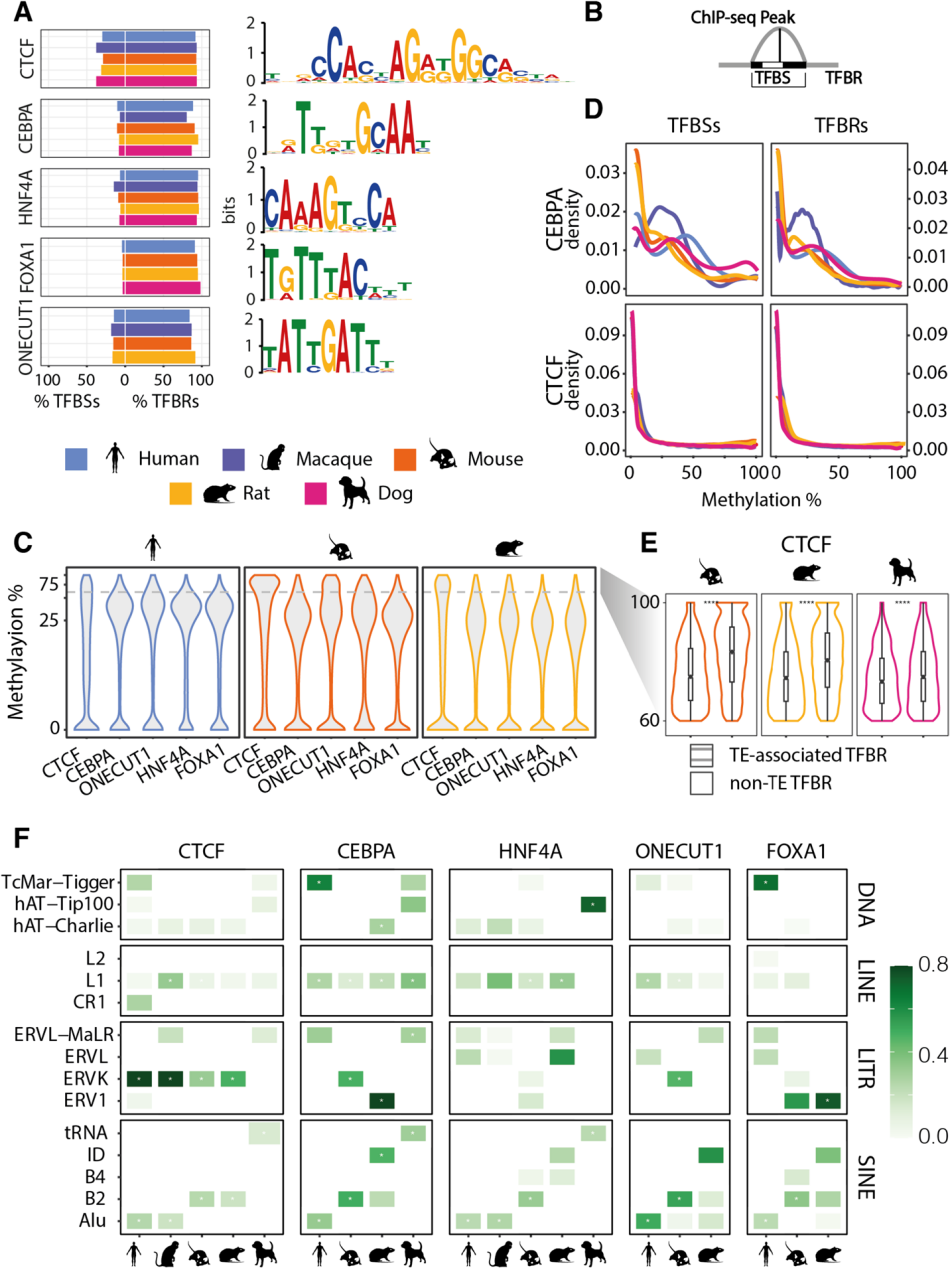
Methylation signatures at TFBRs. **A** Percentage of TFBRs and TFBSs harboring at least one CpG for each TF and species and their binding motifs. Most TFBRs contain CpGs, but rarely at the TF binding site. PWMs calculated from human samples are shown for each TF. **B** Definitions of transcription factor binding regions (TFBRs) and transcription factor binding sites (TFBSs). TFBRs are ChIP-seq peaks, normalized for length within species and TF. TFBSs span the binding motif closest to the ChIP-seq peak summit. **C** Average methylation levels of TFBRs. In all species and TFs, the distributions are bimodal. All TFs have a hypomethylated mode, while CTCF has a higher hypermethylation mode and the remaining TFs have a lower mode. **D** CpG methylation density distributions at TFBRs and TFBSs. All distributions are bimodal, except for CTCF which has unimodal distributions in all species. The hypermethylated regions’ cutoff is marked with a gray dashed (i.e. 60% average methylation). **E** Methylation levels within hypermethylated TFBRs overlapping transposable elements and those that are not repeat-associated. TFBRs have higher 5mC levels when they overlap with transposable elements. **F** Relative positive enrichment of hypermethylated TFBRs versus control hypomethylated TFBRs for selected groups of transposable elements

We explored the presence of CpGs and their methylation levels at the interface between transcription factors and their bound DNA sequences (Fig. 2A). Specifically, we used the ChIP-seq data to first identify transcription factor binding regions (TFBRs) by calling peaks with MACS2 [42] and normalized their length to the average peak length estimated separately for each transcription factor and species (Supplementary Table 2; see the “Methods” section). Transcription factor binding sites (TFBSs) were defined as the DNA sequence where the relevant TF binding motif mapped closest to the ChIP-seq peak summit (Fig. 2B; see the “Methods” section). We found that around 80% of TFBRs harbor at least one CpG, therefore in close proximity to the binding site (Fig. 2A). TFBRs frequently have between three and five CpG sites (Supplementary Fig. S1B). However, only a considerably smaller fraction of regions contains a CpG at the binding site itself (Fig. 2A). CTCF is an exception in that approximately 30% of binding sites contain one or more CpGs, while for the other factors the number of CpGs in the motif range from 3 to 4% for FOXA1, 6 to 14% for HNF4A, 7 to 11% for CEBPA, and 15 to 18% for ONECUT1 (Fig. 2A). This is also reflected in the canonical motif logos—CTCF has more high-scoring CpG instances in the position weight matrix (Fig. 2A, Supplementary Fig. S1A). Taken together, transcription factor binding regions commonly contain CpGs, but most are outside of the binding site itself. This suggests that the closely surrounding region has a few key sites that could be methylated and thus potentially affect binding through direct steric hindrance or interference with a binding partner [11].

The average methylation across TFBRs is a bimodal distribution (Fig. 2C), with both modes below 40% (Supplementary Table 2). This differs from the genomic background bimodal distribution (Fig. 1C) and suggests that most binding regions are either depleted of methylation or have intermediate methylation levels. Intermediate methylation (IM) is also observed when considering CpG methylation density distributions at binding regions and binding sites (Fig. 2D, Supplementary Fig. S1C). These distributions are bimodal and confirm that a considerable fraction of CpGs take up intermediate methylation levels (Supplementary Table 2) and that IM is not simply an artifact of averaging across the regions. Interestingly, hypermethylation occurs more commonly in CEBPA TFBS than in the wider CEBPA TFBR (Fig. 2D and Supplementary Fig. S1E). This supports previous observations that CEBPA may bind to methylated motifs [39], while there is no such evidence for the remaining factors. The macaque CEBPA distribution has a shifted methylation peak, likely caused by the lower quality of the ChIP-seq.

Within its average methylation distribution, CTCF has the same hypomethylated mode as other TFs assayed (0–6% methylation), but a higher hypermethylated mode—around 80% methylation (Fig. 2C, Supplementary Fig. S1D, Supplementary Table2). Given the well-described role of transposable elements (TEs) in driving CTCF binding expansion across mammalian lineages [7, 43], we next investigated if TEs could explain higher average methylation in the hypermethylated mode of CTCF TBFRs. We separated all TFBRs into hypermethylated TFBRs (i.e. with methylation above 60%) and hypomethylated TFBRs (i.e. methylation below 60%), while controlling for ChIP-seq signal strength, CpG abundance, and distance to the closest TSS (see the “Methods” section) and then compared their overlap with TEs. Our analyses show that across all species and TFs, hypermethylated TFBRs overlap TEs significantly more than hypomethylated TFBRs, i.e, for most TFs 20–30% more hypermethylated TFBRs lay in TE sequences (Supplementary Fig. S1F). This is not surprising given the well-documented repression of TEs by 5mC [25]. The rodents’ hypermethylated CTCF binding regions have a stronger association with TEs, i.e. they are 60% more likely to overlap a TE than CTCF hypomethylated TFBRs. We further explored the average methylation distributions of hypermethylated TFBRs by comparing the distributions of TFs associated with TEs to all others (Fig. 2E and Supplementary Fig. S1G). This analysis showed that TE-derived binding regions often have significantly higher hypermethylation modes, especially for CTCF in rodents. Finally, we asked which groups of repeat families contribute to hypermethylated TFBRs more than to the hypomethylated control set (Fig. 2F). CTCF hypermethylated binding regions are enriched in ERVKs in the primate and rodent clades, with Alus in the primates, SINE B2s in the rodents, and SINE-tRNAs in the dog.

Taken together, hypo- and intermediate methylation are signatures of both TFBRs and TFBSs for most TFs, while the hypermethylation signature is unique to CTCF. The wide range of methylation levels we observed most likely reflects the diverse genomic contexts where TF bind, such as transposable elements, promoters, or distal regulatory elements [20].

### Transcription factors bind multiple coexisting DNA methylation profiles

**Fig. 3:**
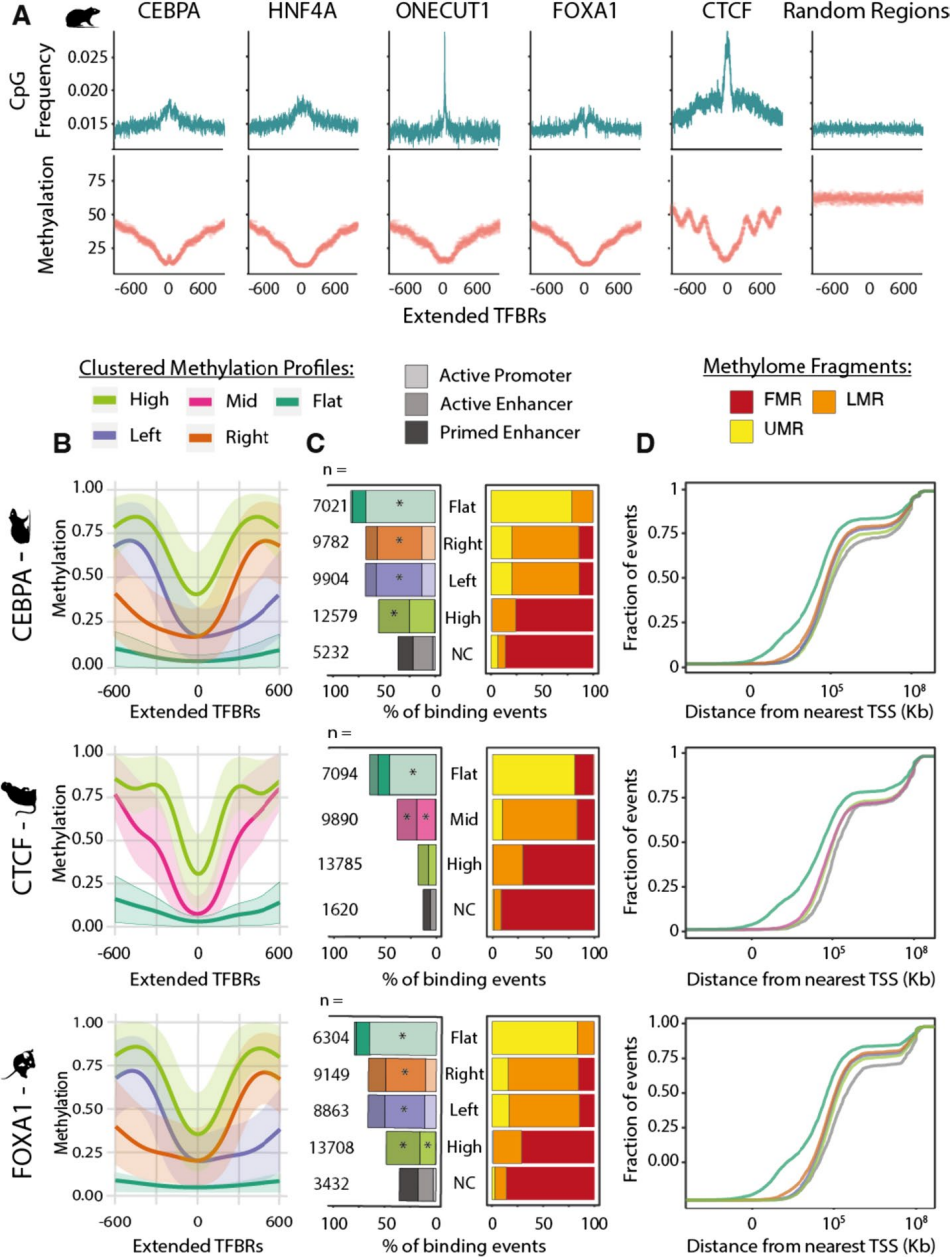
Distinct methylation profiles characterize transcription factor binding regions. **A** Average 5mC and CpG frequency profiles of rat transcription factor binding regions, centered on ChIP-seq peak summits and extended 600 bp on both sides. The number of regions classified in each profile is shown in panel **B**. **B** Clustered 5mC profiles for rat CEBPA, mouse FOXA1, and macaque CTCF binding regions centered on ChIP-seq peak summits and normalized to 1200 bp length. The regions have four types of methylation profiles: “flat” in dark green, “left” and “right” in purple and orange, respectively (both referred to as “specular” in the text), “high” in light green and “mid”, which is unique to CTCF, in pink. **C** Annotations of TF binding regions associated with each clustered methylation profiles defined in panel **B**. On the right, a bar plot showing the percentage of TF binding events belonging to each methylation profile located within Unmethylated (UMRs), Lowly Methylated (LMRs), or Fully Methylated Regions (FMR) of the genome (yellow, orange, and red, respectively). On the left, the percentage of TF binding events in each 5mC profile that are annotated as active promoters, active enhancers, or primed enhancers. The bars are colored according to the methylation profile assignment of the TFBRs and shaded by regulatory element annotation—lightest for active promoters, darker for active enhancers, and darkest for primed enhancers. Asterisks indicate that the annotation category is significantly enriched (*z*-test with Bonferroni correction, *p-values <  < 0.05). **D** Cumulative distributions of the distance of each TF binding region from the nearest transcription start site, grouped by methylation profiles defined in panel B. The* x* axis is in log10 scale

To explore the methylation patterns of the genomic neighborhood bound by transcription factors, we extended the TFBRs to 1200 bp and found that CpG frequency is higher than at random genomic regions and increases approaching the peak summit (Fig. 3A, Supplementary Fig. S2). This is consistent across species and factors, although the width and height of the frequency peaks vary between transcription factors. Though the macaque signal is closer to the background frequency (Supplementary Fig. S2), the same patterns as those seen for other species’ TFs are still evident. Inversely to CpG frequency, methylation levels are high at 600 bp away and then sharply fall at the binding summit (Fig. 3A, Supplementary Fig. S2). This shows that TFBRs are characterized by multiple CpGs in close proximity of the binding site which are predominantly unmethylated when the protein is bound and may be important for TF binding regulation. A few key features stand out from these profiles. First, CTCF exhibits an oscillatory methylation profile which is likely associated to the strong positional pattern of nucleosomes around CTCF binding sites [44, 45]. Second, though CEBPA binding sites have overall low methylation, there is a slight increase in methylation at the binding site. This is consistent with the higher density of hypermethylation in CEBPA TFBSs compared to TFBRs shown in Fig. 2D and Supplementary Fig. S1E. These average profiles demonstrate the most common methylation patterns around transcription factor binding sites. To study the methylation profiles in more detail, we next asked if the average profiles can be further dissected into distinct patterns of local methylation.

To investigate multiple coexisting DNA methylation patterns, we derived the methylation profiles of extended TFBRs using generalized linear model regression and clustering with the *BPRMeth R* package [46]. According to the best-fit model, all extended TFBRs cluster into three or four prototypical methylation profiles with discernible patterns (Fig. 3B and Supplementary Fig. S3; see the “Methods” section). These profiles are very similar across transcription factors and species, and we named them according to their features. The “high” clusters are the most abundant for all factors (Fig. 3C and Supplementary Fig. S4); they have high methylation 300–500 bases from the binding site and show a narrow drop to intermediate methylation levels at the center (Fig. 3B and Supplementary Fig. S3). All transcription factors but CTCF were assigned right and left “specular” profiles (so named because they are mirror images of each other) that have 70% methylation at one end of the profile, a drop to 20% methylation at the binding site, and low methylation maintained to the opposite end of the profile (Fig. 3B and Supplementary Fig. S3). The right and left specular profiles account for about 40% of binding regions (Fig. 3C). The last “flat” profile comprises the smallest group of binding regions (Fig. 3C) and is characterized by wide regions of complete demethylation (Fig. 3B and Supplementary Fig. S3). Only a few thousand binding regions could not be used for clustering due to a small number of CpGs (i.e. less than four) and were hence named non-classified (“NC”). Specular methylation clusters were not observed for CTCF; instead, it has an intermediately methylated cluster, the “mid” cluster. The mid is similar to the high cluster, but with a steeper drop in methylation and complete demethylation at the binding site (Fig. 3B and Supplementary Fig. S3). These prototypical profiles are reproducible across both species and transcription factors (Supplementary Fig. S3). Taken together, clustering classification provides a robust approach to group transcription factor binding regions according to their distinct and conserved methylation patterns.

### DNA methylation profiles associate with different chromatin contexts and functions

DNA methylation levels and CpG density are associated with different regulatory contexts [47]; therefore, we next explored if the prototypical methylation profiles associate with distinct regulatory functions. To test the association with regulatory contexts, we annotated TFBRs using available active promoter (marked by histone 3 lysine 4 trimethylation (H3K4me3) and histone 3 lysine 27 acetylation (H3K27ac)), active enhancer (marked by histone 3 lysine 4 monomethylation (H3K4me1) and H3K27ac), and primed enhancer (H3K4me1 only) calls determined by ChIP-seq [48] (Fig. 3C left side and Supplementary Fig. S4A). To explore their genome-wide methylation context, we annotated TFBRs according to their occurrence in Un-Methylated (UMRs), Lowly Methylated (LMRs), and Fully Methylated (FMRs) Regions ( [31]; see the “ Method” section) (Fig. 3C right side and Supplementary Fig. S4B).

A significantly high proportion (around 70%) of TFBRs with the flat profile overlap with active promoters (Fig. 3C left side and Supplementary Fig. S4A). These are mostly found within UMRs, very close to transcription start sites (TSSs), and 35% overlap an annotated TSS (Fig. 3D and Supplementary Fig. S5B). In fact, many of these regions are also found within CpG islands (Supplementary Fig. S6B). This is consistent across all species and for all transcription factors and shows that TFBRs of the flat clusters are largely promoter regions. The right and left specular profiles comprise similar numbers of binding regions (Fig. 3C), have comparable methylation levels (Supplementary Fig. S7A) and similar annotations (Fig. 3C, D, Supplementary Figs. S4 and S5), clearly underlying the same regulatory contexts. To explore the influence of transcription on the directionality of the specular profiles, we further explored those overlapping TSSs and found that the left profiles mostly associate with transcription on the forward strand, while the right associates with transcription on the reverse strand (Supplementary Fig. S5A), suggesting that the unmethylated regions correspond to the first exon of active genes. Therefore, for further strand-agnostic analyses, we grouped these two profiles together. Most TFBRs of the specular profiles are significantly found in LMRs and enhancers, with only a small fraction overlapping active promoters. Similarly, the high profile comprises TFBR significantly overlapping enhancers, but predominantly in fully methylated regions (FMRs). The non-classified (NC) TFBRs are found in FMRs and about 40% of them were annotated as enhancers. The enhancer regions of the specular, high, and NC groups are equally far from TSSs (Fig. 3D and Supplementary Figure S5B) and do not overlap with CpG islands (Supplementary Fig. S6). Generally, TF binding events associated with the different methylation profiles show similar binding intensities, measured by the ChIP-seq signal fold enrichment distributions (Supplementary Fig. S7). However, binding events associated with the high profiles had significantly lower fold enrichment scores than the specular profiles (Supplementary Fig. S7). Next, we compared CpG densities associated with the methylation profiles and found that flat profile TFBRs are the richest, while those with the high profile have significantly fewer CpGs (Supplementary Fig. S6A), showing that the profiles have unique CpG densities. We show that prototypical DNA methylation profiles distinguish between unmethylated CpG-rich promoter regions, lowly methylated enhancers with intermediate CpG density, and highly methylated CpG-poor enhancers.

CTCF has exceptionally few overlaps with regulatory regions, except for TFBRs of the flat profile which have comparable annotations to the other transcription factors. 25% of TFBRs with the mid profile, found only for CTCF, and 10% with the high profile overlap primed or active enhancers. This overlap is too small to confidently assign CTCF TFBRs of these profiles to regulatory contexts. Moreover, TFBRs with a mid-profile typically show higher binding intensity (Supplementary Fig. S7). Given that CTCF not only has a role in regulation, but also in genome stability and architecture, the low overlap with promoters and enhancers likely reflects methylation landscapes of alternative chromatin contexts.

Taken together, transcription factors bind within different chromatin contexts that are marked by specific DNA methylation profiles which are deeply conserved in mammals.

### DNA methylation levels are coupled to transcription factor binding divergence

**Fig. 4:**
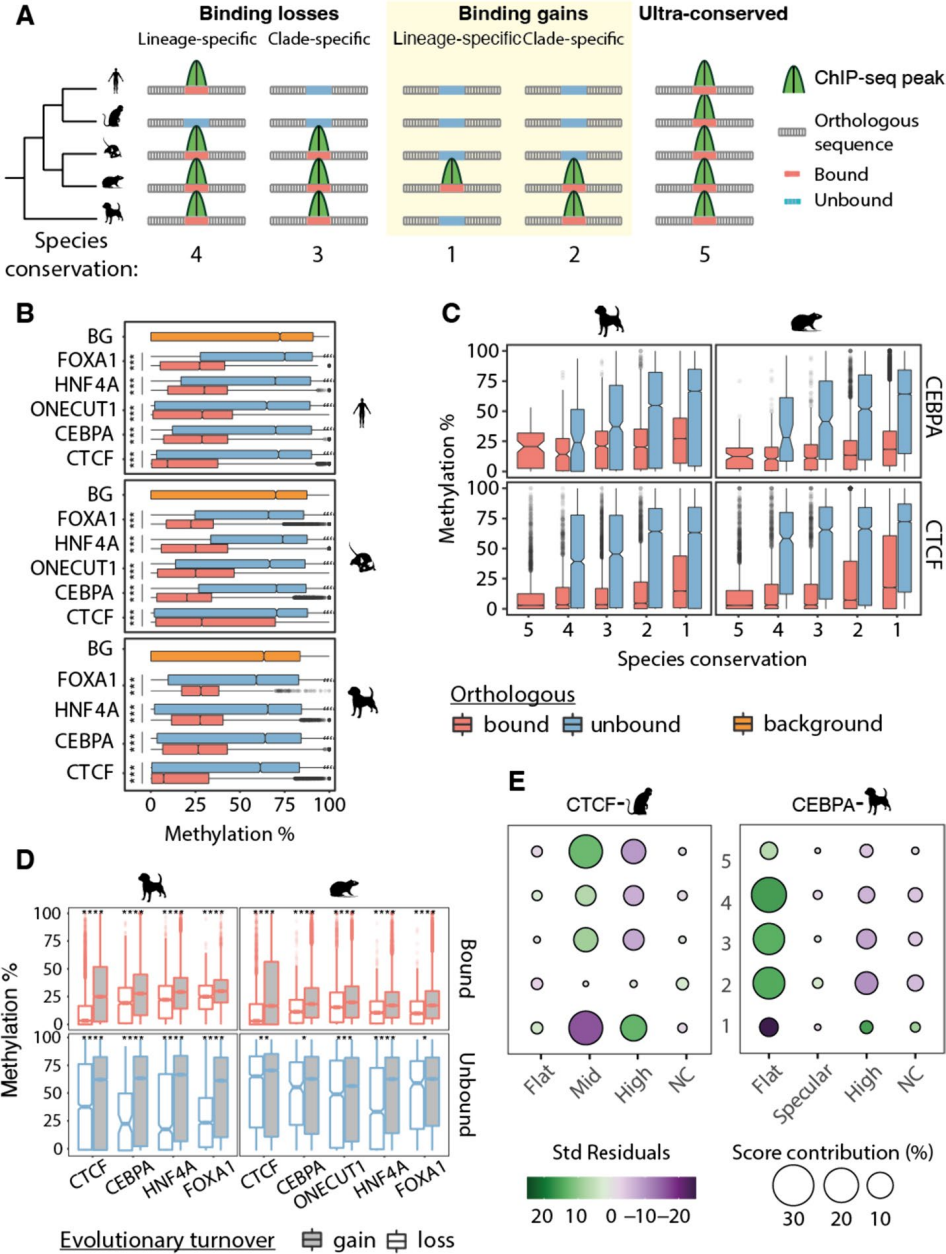
Coevolution of methylation and TF binding in mammals. **A** Schematic representation of the phylogenetic parsimony approach (adapted from [10]) to define species conservation categories and number of species with binding conservation. Briefly, TF binding events were first aligned and compared across species, then divided using parsimony in lineage- and clade-specific binding losses, and lineage- and clade-specific binding gains. Regions with experimentally determined binding in the species were called orthologous bound, and those without binding unbound. Ultra-conserved binding events were defined as those bound across all species. Below, examples of corresponding degrees of species conservation defined by the total number of species that share a TF binding event. **B** Average 5mC level distribution of orthologous bound regions, orthologous unbound regions, and genomic background (BG), with significant differences marked with asterixis (Wilcoxon test with Bonferroni correction, ***p-value ≤ 0.001). **C** Average 5mC distributions within CEBPA and CTCF orthologous bound and orthologous unbound regions divided by species conservation categories defined in panel A (Jonckheere-Terpstra trend test, *p*-values < 2.2e10^6^), shown for dog and macaque. **D** Average 5mC distributions at orthologous bound and unbound regions of dog’s and rat’s TFBRs, further divided into evolutionary binding loss and gain events according to our parsimony approach. Orthologous sequences that concur in the definition of clade- or lineage-specific losses or gains are compared based on the presence (bound) or absence (unbound) of a binding event. Orthologous sequences defining a binding gain consistently have higher methylation levels than binding losses, both when unbound and bound by TFs (Wilcoxon test with Bonferroni correction, ***p-value ≤ 0.001). **E** Relationships between species conservation and 5mC profiles. Balloon plots show standardized residuals from an association analysis (chi-square test of independence) between 5mC profiles and TF binding conservation categories for dog’s CEBPA and macaque’s CTCF TF binding events. Positive residuals indicate a positive association between the degree of species conservation and methylation profile, while negative residuals indicate negative associations. For example, dog’s CEBPA binding events with a flat profile are positively associated with higher levels of species conservation, while they are negatively associated with lineage-specific binding events. The size of the balloons is proportional to the percentage of contribution to the total Chi-square score, therefore highlighting the most influencing combination of species conservation and methylation profile to the overall statistics

We investigated the evolutionary conservation of DNA methylation patterns across species and its association with transcription factor binding divergence. First, we leveraged the EPO multiple species alignments from Ensembl version 98 [49] to define regions orthologous to TFBRs by projecting their coordinates onto the other species’ genome (Fig. 4A; see the “Methods” section). Next, we compared DNA methylation levels across TFBRs and their orthologous counterparts to check for TF binding in the orthologous region. Typically, orthologous regions that are not bound by a TF have higher methylation levels than those that do, with average medians of around 75% and 20%, respectively (Fig. 4B). The orthologous unbound regions have hypermethylation levels comparable to randomly selected genomic segments (Fig. 4B) and to those previously described for the non-regulatory portion of the genome [28]. We further assessed whether the number of species with binding affects the methylation in the orthologous unbound region (Fig. 4C) and observed a general increase in methylation of the unbound regions when fewer species retain the binding activity, while methylation in the bound regions remains consistent regardless of the conservation (Fig. 4C).

We leveraged the parsimony principle and the structure of our phylogenetic tree to subset orthologous regions into evolutionary binding losses and gains (Fig. 4A; see the “Methods” section). We found that TF binding events classified as an evolutionary loss have lower overall methylation than gains of binding, even when controlling for the binding state of the orthologous site (Fig. 4D and Supplementary Fig. S8). However, the difference between methylation levels of bound and unbound orthologous regions is more pronounced for binding losses than binding gains (Supplementary Fig. S8A). These results show coordinated evolution of DNA methylation and TF binding and suggest that orthologous regions which lost a binding event over evolution are reset back to the hypermethylated levels of the non-regulatory genome.

We further investigated whether there is a correlation between DNA methylation and the degree of TF binding conservation (Fig. 4A). Lineage-specific binding events (i.e. those bound in only one of the species studied) have intermediate levels of methylation; as the number of species sharing a binding event at orthologous locations increases, the methylation level decreases (Fig. 4D, Supplementary Fig. S8B). DNA methylation is inversely correlated to the degree of species conservation, even for orthologous unbound regions. These results show that DNA methylation co-evolves with the binding divergence of specific TFs.

Next, we explored the association of DNA methylation profiles across TFBRs with different degrees of binding conservation. Specifically, we performed a chi-square test of independence between the DNA methylation profiles of TFBRs and evolutionary conservation and found a significant overall association (*p*-value <  < 0.05; Fig. 4E and Supplementary Fig. S9). A closer investigation of the individual residual values showed that the high profiles contributed strongly to the overall dependence statistic for all TFs. Lineage-specific binding events had a positive association, while higher degrees of conservation contribute negatively. The flat profiles contributed to all TFs except CTCF with the opposite association: higher species conservation was negatively associated. CTCF instead had high residual values for mid-methylation profiles, with the same trends with species-conservation association as the flat profiles of other TFs. These results show that methylation profiles are subject to different evolutionary pressures, as they distinctly associate with different levels of species conservation.

In conclusion, DNA methylation is coupled with TF binding divergence at different levels. Bound regions are more highly methylated than orthologous unbound regions. Furthermore, the methylation of both bound and unbound regions tracks with the degree of species conservation. Finally, different methylation profiles are associated with high and low species conservation, indicating that their regulatory contexts might contribute to the evolutionary coupling of TF binding and DNA methylation.

## Discussion

To explore the co-evolution of DNA methylation and TF binding, we combined newly generated bisulfite-sequencing experiments and matched publicly available ChIP-sequencing data for five transcription factors in five mammals. These datasets allowed us to determine the spatial variation of DNA methylation across transcription factor binding regions and characterize the genomic contexts that establish distinct DNA methylation patterns. We leveraged interspecies differences that arose over 96 million years of evolution among the five species and revealed coordinated evolution between transcription factor binding divergence and DNA methylation patterns.

### CpG methylation levels in transcription factor binding regions depend on the genomic context

The extent of DNAm’s role in modulating TF binding through changes of affinity towards their target sequences is still not clear [36, 50]. Our data show that only a small subpopulation of TFBSs contain a CpG and thus could be directly affected by 5mC. Considering the instability of methylated cytosines [51], this suggests that CpGs may be generally negatively selected at TFBSs of the studied TFs and those present could be protected from mutagenic processes through other mechanisms, but further investigation is necessary to confirm this hypothesis.

The wider genomic context surrounding the investigated TFBRs more often harbor CpGs than their TFBSs. Their methylation state is less likely to directly disrupt the TF binding site; however, it can still affect binding through processes such as steric hindrance or the recruitment of chromatin remodelers. Our results therefore suggest that local demethylation in TFBRs of the studied TFs is rarely due to the direct competition between transcription factors and DNAm levels [20, 52]. Furthermore, the intermediate methylation and complete demethylation that we observed at TFBRs are consistent with a recently published model describing distinct methylation dynamics between different genomic contexts [30]. For example, it showed that intermediate methylation at distal regulatory regions is the result of an increased rate of passive demethylation and variable rates of de novo methylation.

To enhance interpretability across genomic contexts, we further described local methylation patterns within TFBRs and their genomic surroundings using generalized linear model regression and clustering [33, 46]. We revealed that methylation patterns of TF binding regions can be summarized in three prototypical profiles and reflect their genetic and chromatin contexts. The profile with low levels of methylation throughout (i.e. flat) was typical of CpG-rich promoter regions and is likely the result of H3K4me3’s inhibition of de novo methylation [53]. On the other hand, profiles with intermediate levels overall were enriched within distal regulatory elements marked by H3K4me1. The high profiles had intermediate to high methylation and mostly occurred in CpG-poor enhancers. The specular profiles had low to intermediate methylation and were also marked by the active histone mark H3K27ac. Thus, different types of regulatory regions can be discriminated solely based on 5mC patterns.

Taken together, our results suggest that local methylation levels are determined through competition among a wider number of context-dependent regulatory players such as transcription factors, chromatin remodelers, and DNA methylation effectors.

### Coevolution of 5mC and transcription factor binding

Although most transcription factors bind extremely conserved DNA motifs in mammals, their genome-wide binding patterns are highly divergent between species [3, 10]. Our study reveals that DNAm follows inter-species divergence of cis-regulatory activity. Specifically, 5mC levels are low at TF-bound regions, but they increase at orthologous locations after binding loss to levels of non-regulatory active intergenic CpGs. Given that DNA methylation broadly mimics the occupancy of various TFs [12, 54], the detected gains of methylation may be indicative of complete regulatory turnover of the orthologous region.

Within each genome, we showed that 5mC levels are inversely proportional to the number of species with conserved binding. This is true for all orthologous regions regardless of whether they are bound, though unbound regions have higher methylation values on average. The methylation differences are prominent even for factors that rarely have CpGs in their motifs suggesting that regional methylation changes may be associated with loss of binding partners or other disruptions to the regulatory landscape. These results can partly be explained by enhancers evolving more rapidly than promoters [55, 56]: binding sites with flat methylation profiles characterize promoters and have low within-species methylation, while those with high methylation profiles characterize enhancers and have higher within-species methylation.

We show that the genomic context partially explains the evolutionary relationship between 5mC and TF binding divergence, but more elaborate models are needed to define the rate of DNAm turnover within these contexts.

Notably, CTCF’s 5mC profiles evolve differently than those of the other TFs. Specifically, the flat CTCF profile, despite being enriched with promoters, is not associated with high species conservation. This may be due to the previously described redundancy of CTCF near functionally important sites [17] or context-effects determinants of CTCF occupancy [57], which may buffer the loss of a CTCF binding event in one species through turnover. This suggests that CTCF’s binding events within the flat cluster are under less stringent evolutionary pressure than the wider promoter region. On the other hand, the CTCF-specific methylation profile (i.e, mid) is strongly associated with high species conservation, depleted at lineage-specific binding events, and may be subjected to high evolutionary pressure. The mid profile might correspond to the subset of CTCF sites that have CpGs in their binding sites and are methylation sensitive [58]. This points to an important role for these binding sites, but further work is necessary to characterize their function and features.

Our study details an association between DNA methylation and TF binding in the evolution of transcriptional regulation. Although relatively few TFs have CpGs amenable to DNA methylation within their core binding motifs, these binding sites often feature CpGs in the wider binding regions. The significant differences in methylation levels between bound and unbound orthologous regions across species indicate that methylation likely influences TF binding through cooperative partners or higher-order chromatin structure. These results suggest that changes in DNA methylation are at least part of the reason why transcription factor binding changes rapidly across species, even without changes to the core TF binding motif.

## Methods

### Publicly available data

All ChIP-seq data are publicly available and were retrieved from ArrayExpress (https://www.ebi.ac.uk/arrayexpress). CTCF ChIP-seq data for all species can be downloaded under accession number E-MTAB-437. HNF4A, ONECUT1, FOXA1, and CEBPA ChIP-seq data for all species can be retrieved under accession number E-MTAB-1509. We used all the available experiments except ONECUT1 from dog, due to the lack of replicates. ChIP-seq of histone modifications and processed regulatory region calls can be accessed in ArrayExpress with accession number E-MTAB-7127.

### Tissue preparation

Mammalian liver samples were extracted post-mortem, perfused with PBS, and flash-frozen in liquid nitrogen. Tissues were prepared immediately post-mortem (typically within an hour) to maximize experimental quality and were kept on ice until processed to minimize potential DNA degradation. Total genomic DNA was extracted from each sample with commercial reagents and following manufacturer guidelines (Qiagen, DNAEasy Blood&Tissue kit). Details on origin, number of replicates, sex, and age for each species’ sample are in Supplementary Table 1.

DNA from at least two independent biological replicates from different animals was prepared for each species. Wherever possible, livers from young adult males were used. Samples of healthy liver tissue from humans were obtained from the Addenbrooke’s Hospital at the University of Cambridge under license number 08-H0308-117 “Liver specific transcriptional regulation”. Mouse samples were obtained from the Cambridge Institute under Home Office license PPL 80/2197.

### Whole-genome bisulfite sequencing (WGBS) protocol

Mammalian DNA was subjected to bisulfite conversion using the Epimark CT conversion kit using Agilent and/or Epimark polymerase (Supplementary Table 1). Subsequently, libraries were prepared using the NEBNext Ultra DNA library preparation kit and sequenced using an Illumina massively parallel sequencer.

### Genome resources

All genomes were downloaded from the Ensembl ftp version 98 [59] as top-level assembly files. We then filtered out patches and scaffolds and retained only assembled chromosomes. The species genome versions used are the following: GRCh38.p13 for human, GRCm38.p6 for mouse, Mmul_10 for macaque, Rnor_6.0 for rat, and CanFam3.1 for dog.

### WGBS data processing

Paired-end FASTQ files were trimmed and adapters removed using *TrimGalore!* version 0.6.4_dev [60] with default parameters. We then processed the data using *Bismark* version 0.22.3 [61]. First, we performed in silico bisulfite conversion of the reference genome, i.e., C → T and G → A conversions, using the *bismark_genome_preparation* script. Next, reads were mapped to each species’ genome by running *bismark* with default parameters. Duplicate reads were removed from bam files with *bismark_deduplicator*, before extracting methylation calls using *bismark_methylation_extractor* with the following parameters: *bismark_methylation_extractor –comprehensive –merge_non_CpG –bedGraph –no_overlap –ignore_r2*. Finally, we generated a coverage file using the script *coverage2cytosine* with the following parameters: *coverage2cytosine –merge_CpG –zero_based*. Methylation calls were considered in downstream analyses only if supported by methylation evidence from at least four CpGs (i.e., minimum four read coverage).

### ChIP-seq data processing

Paired-end FASTQ files were trimmed and the sequencing adapters removed using *TrimGalore* [60]*!* with default parameters. Trimmed reads were then mapped to each species’ genomes using *bowtie2* version 2.3.5.1 [62] with default parameters. We next called peaks using *MACS2* version 2.1.4 [42] using the narrow peak mode and the *-f BAMPE* parameter. FOXA1 experiments from macaque were removed from further analyses due to a smaller number of peaks called compared to the other species. To call reproducible peaks, we found overlap between replicates’ peaks with *bedtools intersect* v2.29.2 [63] and kept those that overlap with at least one base between both replicates. For further analyses, we represented the reproducible peak as the original replicate peak with the strongest signal, as defined by the *p*-value.

### Defining transcription factor binding regions (TFBRs) and their methylation coverage

To define transcription factor binding regions (TFBRs), we normalized reproducible peak sets for length. Specifically, we extended the reproducible peaks from the peak summit equally in both directions until we reached the average total peak length within that species and factor. To calculate the number of CpGs that overlap with TFBRs, we used *bedtools intersect* and *bedtools groupby* to intersect the TFBRs with the methylation coverage file. We repeated the same process to calculate the average methylation level associated with TFBRs, but only considered CpGs covered at least four times. Average methylation level distributions within TFBRs were tested for unimodality in *R* with the *mod.test* function from the *multimode R* package (v 1.5). We identified TFBRs with intermediate or hypermethylation levels as TFBRs with average methylation values ± 15% from the highest mode. Specifically, CTCF had a bimodal distribution and its highest mode was within hypermethylated values, while all other TFs had a second mode within intermediate methylation.

### Motif discovery and transcription factor binding site (TFBS) annotation

Motif discovery was conducted with the *MEME suite* version 5.0.5 [64]. From each peak set, we selected the 500 strongest peaks, i.e., with the lowest *MACS2* *p*-values, and restricted them to 100 bp centered on the peak summit. From this representative set, we performed de novo motif discovery for the most significant motif using *MEME* with the following parameters *-mod oops -dna -revcomp -nmotifs 1*. Next, we identified motif matches to these newly generated motif position weight matrices (PWMs) in each TFBR set with *FIMO* using a *p*-value threshold *-thresh 0.005* and the option *-max-stored-scores 1000000000*. To define transcription factor binding sites (TFBSs), we kept the motif closest to the peak summit. We calculated the number of CpGs and average methylation levels within TFBSs with the same procedure as for TFBRs.

### Transposable elements’ overlap with TFBRs

We made the hypermethylated test TFBRs set by selecting those with average methylation levels equal to or greater than 60%. To ensure ample sample size, we filtered out any combination of species and TF with less than 200 hypermethylated TFBRs. Next, we created a matched control set of TFBRs with average methylation below 60%, but with an equal distribution of CpGs, ChIP-seq fold enrichment values, and distance to the closest TSS. To do so, we used the *MatchIT* v4.5 library in* R *with a caliper option (0.001) to prune unmatched TFBRs. We overlapped test and control TFBRs with transposable element sequences annotated in repeat masker output files from [47] using *bedtools intersect* with default parameters. The difference between the proportions of TFBRs overlapping any TE in the test and control set was tested in* R *with the *Z*-test implemented in the *prop.test* function, and the heatmap in Supplementary Fig. S1F was generated using *ggplot*.

Relative enrichment of specific TE groups between test and control TFBRs was calculated as follows. For each species and TF, we used the class column of RepeatMasker to annotate TFBRs with class (i.e., DNA, LINE, LTR, SINE) and subgroup classification (e.g., L1, ERVK, B2) as defined by RepBase. Next, within each class of TE within hypermethylated and control TFBRs, we calculated the fraction belonging to each subgroup. To calculate relative enrichment, we calculated the log2 transformation between hypermethylated and control TFBRs’s fractions. For example, we calculated the percent of all LTR-overlapping hypermethylated TFBRs overlapping an ERVK, divide it by the percent of control LTR-overlapping TFBRs, divided these two values, and log2 transformed them. The resulting value represents the relative enrichment of ERVK in hypermethylated TFBRs compared to the test set, where positive values indicate that the TE is more enriched in the hypermethylated TFBRs than in the control set. *P*-values were calculated in *R* with the *Z*-test implemented in the *prop.test* function. The heatmap in Fig. 2F was filtered for negative values to show only those TEs that preferentially associate with hypermethylated TFBRs.

### 5mC and CpG profiles

We modeled 5mC profiles with an average methylation approach and used the probabilistic model implemented in the *BPRMeth R* package [46]. We first extended TFBRs to 2 Kb centered on the ChIP-seq summit, then intersected these extended regions with methylation coverage files.

To calculate average 5mC profiles (Fig. 3A and Supplementary Fig. S2), we first created a matrix in *R* version 4.0.1 [65] where each row is an extended TFBR and the positions denote the presence and methylation levels of CpGs covered at least 4 times. For each column, we calculated the average methylation level and plotted the results using *ggplot2 jitters* version 3.3.4 [66]. To calculate CpG frequency, we repeated the same process but used all CpGs, regardless of coverage.

To model and cluster 5mC profiles with a probabilistic approach (Fig. 3B and Supplementary Fig. S3), we inferred profiles using the mean-field variational inference (Variational Bayes) method from the *BPRMeth* *R* package v1.8.2 [46]. For each species and each transcription factor, we independently optimized the number of radial basis functions (RBFs)—which determine the spatial resolution of the methylation profiles—and the number of clusters. To do so, we used the Bayesian Information Criterion (BIC) and set the number of clusters to three for CTCF and to four for the remaining transcription factors. The combination of parameters selected for each species and transcription factor is shown in Supplementary Fig. S3.

### Functional categories of TF binding regions

We further annotated TFBRs into functional categories using regulatory region calls from [48], CpG Islands (CGIs), and methylome segment annotations. CpG islands were calculated for each species’ genome with the *EMBOSS cpgplot* v6.6 [67] using default parameters. To segment the methylome in UnMethylated Regions (UMRs), Lowly Methylated Regions (LMRs), and Fully Methylated Regions (FMRs), we used the *MethylSeekR R* package version 1.22 [31], setting the FDR cutoff to 5 and the *m* parameter to 0.5. To make the assignments, TFBRs were overlapped with each functional category above using *bedtools intersect* v2.26 (1 bp overlap required)*.* In Fig. 3C (left side, Supplementary Fig. 4B), we show the distribution of TFBRs’ clustered profile assignments between these functional categories. Finally, we defined transcription start sites (TSSs) as the start of every annotated transcript in GTF files downloaded from the Ensembl version 98 ftp [59]. We used *ggplot2* to plot cumulative distributions of the distance between TFBRs and their closest TSS (Fig. 3D and Supplementary Fig. S5).

### Evolutionary conservation of TF binding regions

To make evolutionary conservation calls for TFBRs, we used their overlap in whole genome alignments. Specifically, we used the EPO eutherian mammal alignment from Ensembl version 98 [49] to align each TFBR with every other species’ genome and extract the genomic coordinates of these orthologous sequences. Next, we overlapped the orthologous coordinates with transcription factor binding locations of the corresponding TF from the species projected to, and if overlap was found, we called the binding conserved. For example, CEBPA binding locations from mouse were first aligned to the rat genome, then the aligned orthologous locations on the rat genome were intersected with CEBPA binding locations from rat. If the projected sequence and the rat CEBPA binding region overlapped with at least 1 bp, these were considered conserved between mouse and rat. For each binding event within each species, we then summarized the number of species the binding sequence was alignable to and the number of species the binding sequence was both alignable and conserved.

We categorized binding events in two ways (Fig. 4A), the first according to the number of species with conserved binding and the second according to phylogeny. The number of species with conservation was defined irrespective of phylogeny; for example, if the binding event was shared at orthologous locations in exactly three species, that was called a 3-way conserved binding event. The second categorization was based on the phylogenetic relationships between the species. Specifically, we considered binding only shared by mouse and rat exclusive to the rodent clade and binding only in human and macaque exclusive to the primate clade. We further built on the phylogenetic approach using the parsimony method defined in [10] to call binding events ultra-conserved (if the binding event is shared by all species studied), lineage-specific binding loss (if the binding event is present in all species except one), lineage-specific binding gain (if the binding event is present only in one species), clade-specific binding gain (if the binding event is present in only one clade), and clade-specific binding loss (if the binding event is present in all other species except one clade).

To explore the effect of methylation levels on binding conservation, we intersected the orthologous sequences on each species’ genome with the corresponding methylation coverage files to obtain the number of CpGs and their methylation levels, regardless of whether the corresponding TF was bound in that species. In Fig. 4B, C, and Supplementary Fig. S8A, we calculated average 5mC levels for the bound and unbound sequences within each evolutionary category. For example, within a rodent-specific binding gain category (also defined as a 2-way binding event), the bound regions correspond to the orthologous regions where a TFBR was identified in mouse and rat, while the unbound regions correspond to the orthologous locations in the remaining species not bound by the TF.

### Association between 5mC profiles and evolutionary conservation

To test for independence between evolutionary categories of binding conservation and clustered methylation profiles, we first used them to create a contingency table in *R* and then performed a chi-squared test using the *chisq.test* function. We extracted the chi-square standardized residuals and calculated each cell contribution to the chi-square score as the squared chi residuals over the chi statistics, then multiplied by 100. In Fig. 4E and Supplementary Fig. S10, the results are plotted as balloon plots with *ggballoonplot* from the ggpubr* R* library [68].

### Supplementary Information

**Additional file 1: Supplementary file 1**. All supplementary figures related to this manuscript.

**Additional file 2:  Supplementary Table 1**. Description of the WGBS experiments performed in this study, including the unique identifiers for each library and tissue sample collection details for each species.

**Additional file 3: Supplementary Table 2**. Length - the normalized peak lengths for each transcription factor and species; modes – antimode, lowest and highest modes of the average methylation level distributions within TFBRs; modes - fraction of TFBRs classified as hypermethylated or intermediately methylated.

**Additional file 4: Additional Data 1**. Additional details on CEBPA and ONECUT1 binding events. A comprehensive table including transcription factor binding coordinates, genomic and 5mC profiles annotations and their cross-species alignability and conservation.

**Additional file 5: Additional Data 2**. Additional details on CTCF and FOXA1 binding events. A comprehensive table including transcription factor binding coordinates, genomic and 5mC profiles annotations and their cross-species alignability and conservation.

**Additional file 6: Additional Data 3**. Additional details on HNF4A binding events. A comprehensive table including transcription factor binding coordinates, genomic and 5mC profiles annotations and their cross-species alignability and conservation.

**Additional file 7: Additional File 7**.Peer Review History

## Data Availability

The WGBS datasets generated during the current study are available in the ArrayExpress repository with accession number E-MTAB-11946 (https://www.ebi.ac.uk/arrayexpress/E-MTAB-11946) [69]. The ChIP-seq datasets analyzed during the current study are available in the ArrayExpress repository with accession numbers E-MTAB-437 (https://www.ebi.ac.uk/arrayexpress/E-MTAB-437) [70] and E-MTAB-1509 (https://www.ebi.ac.uk/arrayexpress/E-MTAB-1509) [71]. The source code is available on github at https://github.com/Mavti/evoDNAm_paper and is deposited on zenodo under 10.5281/zenodo.7756200.
