# Supplementary material for: DNA methylation patterns of transcription factor binding regions characterize their functional and evolutionary contexts
Source: Genome Biol. 2024 Jun 6;25:146. doi: 10.1186/s13059-024-03218-6 (PMC11155190; doi:10.1186/s13059-024-03218-6)
Supplement: Supplementary file 1 — Additional file 1: Supplementary file 1. All supplementary figures related to this manuscript. [file 13059_2024_3218_MOESM1_ESM.docx]

**
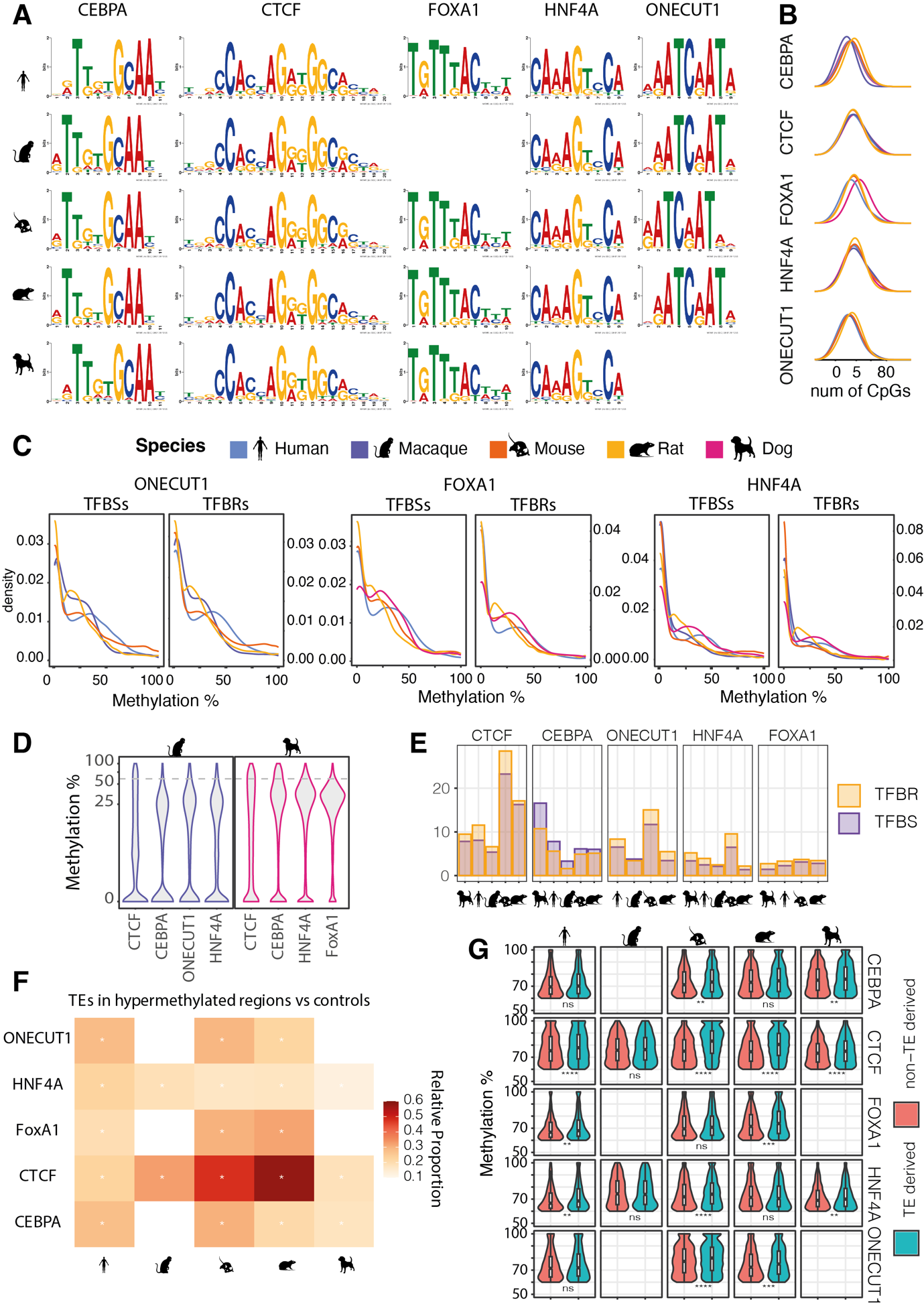
**

**Figure S1**: **A)** Position weight matrices (PWMs) calculated de novo for all transcription factors in this study and in all species. **B)** Density distributions of number of CpGs within TFBRs. TFBRs frequently harbor between 3 and 5 CpGs. The X axis is in log scale. **C)** CpG methylation density distributions at TFBRs and TFBSs for ONECUT1, HNF4A and FOXA1 in all species. **D)** Average methylation distributions within TFBRs in macaque and dog for all TFs. **E)** Overlaid bar plots showing the percentage of TFBRs and TFBSs with average methylation above 65%. For all TFs except CEBPA, the percentage of TFBRSs with high methylation exceed that of TFBSs. **F)** Heatmap showing the relative proportion of hypermethylated TFBRs overlapping TEs versus control hypomethylated TFBRs. Asterisks indicate statistical significance (Z-test, p-value << 0.05) **G)** Methylation levels within hypermethylated TFBRs overlapping transposable elements and those that are not repeat associated (Wilcoxon test with Bonferroni correction, *** = p-value <= 0.001).

**
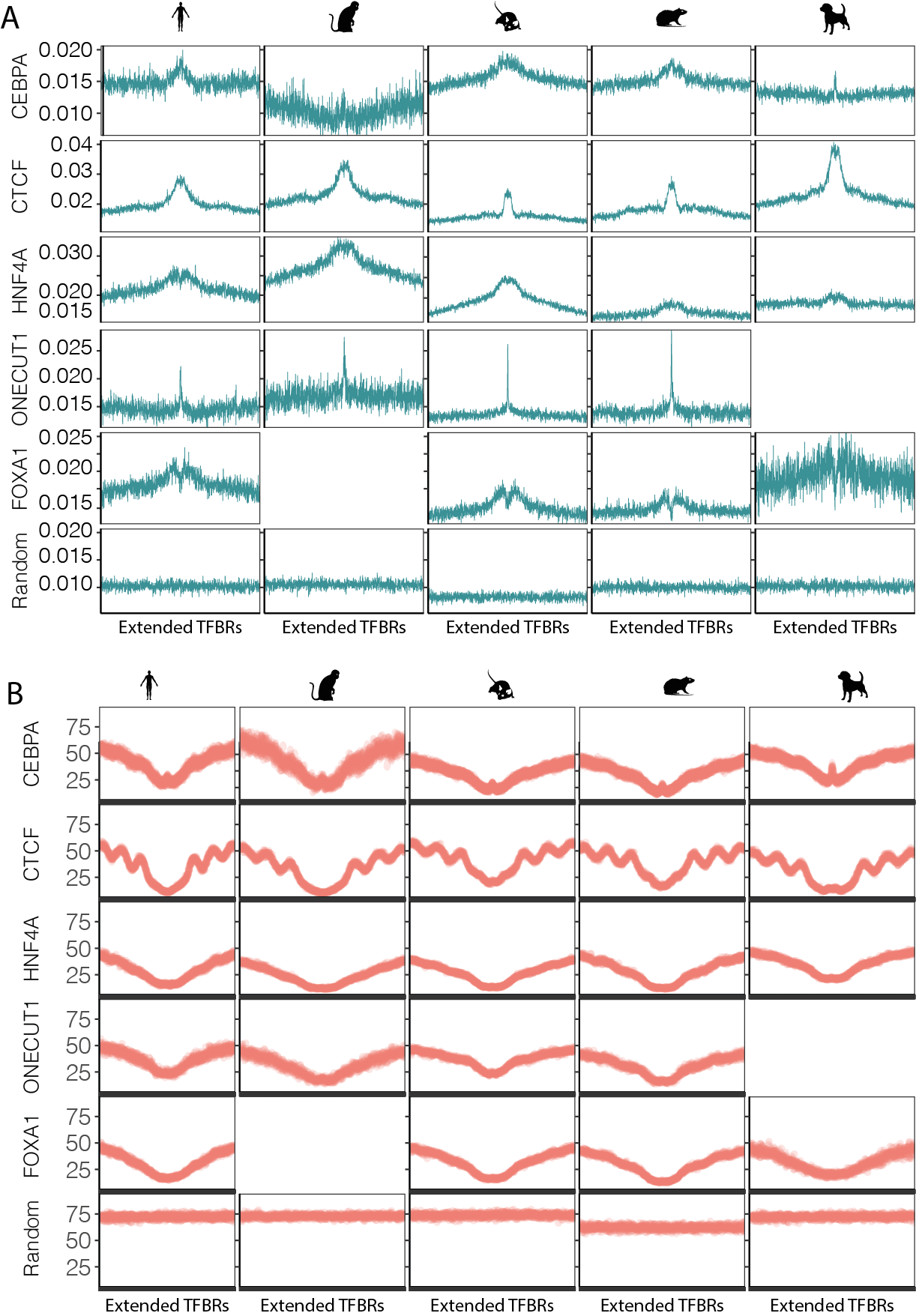
**

**Figure S2**: Average CpG frequency (panel A) and 5mC profiles (panel B) at 1200bp wide transcription factor binding regions, centered on ChIP-seq peak summits for all species.


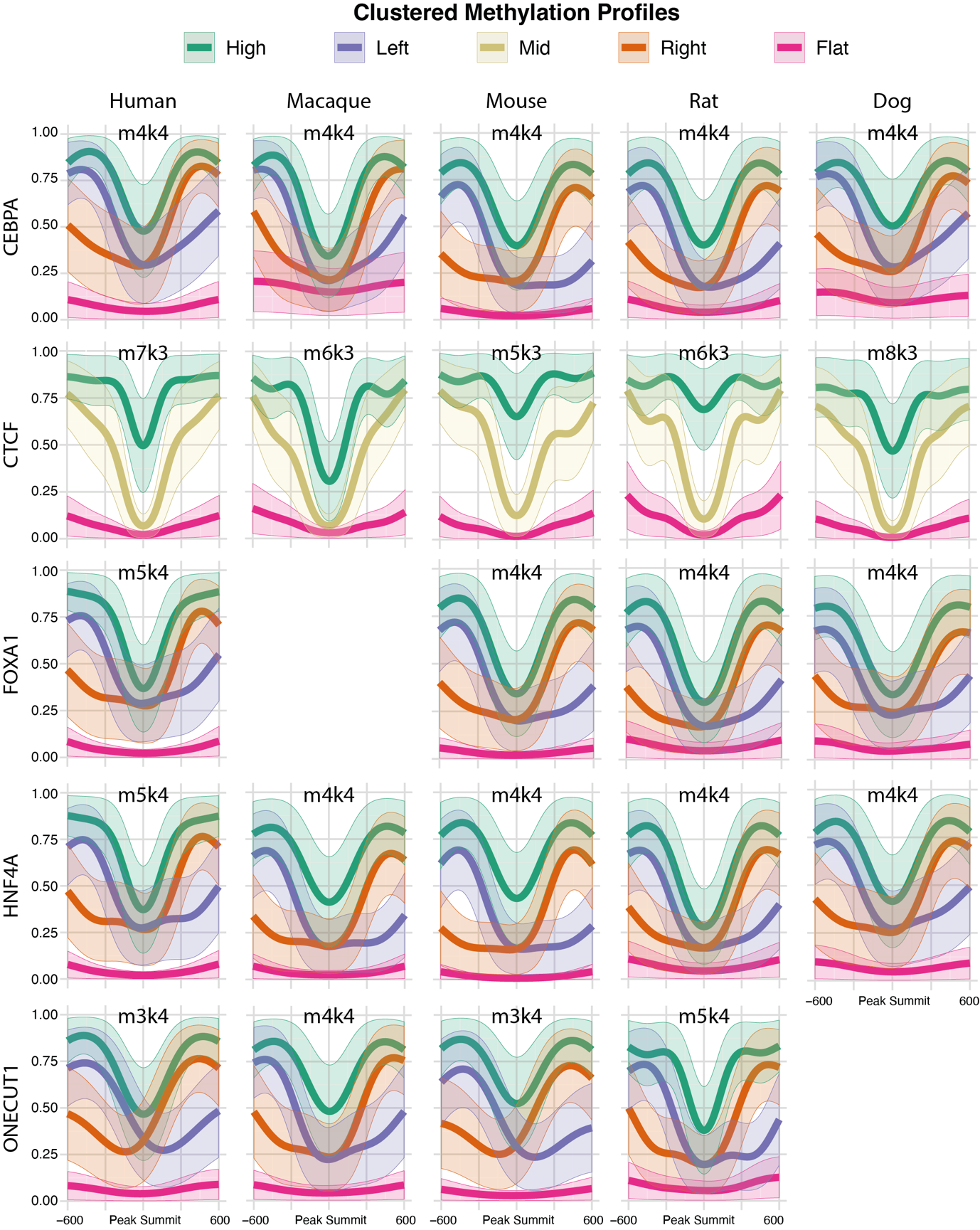


**Figure S3**: Clustered 5mC profiles for all species and TFs. Above each plot, the optimized M and K parameters are reported.

**
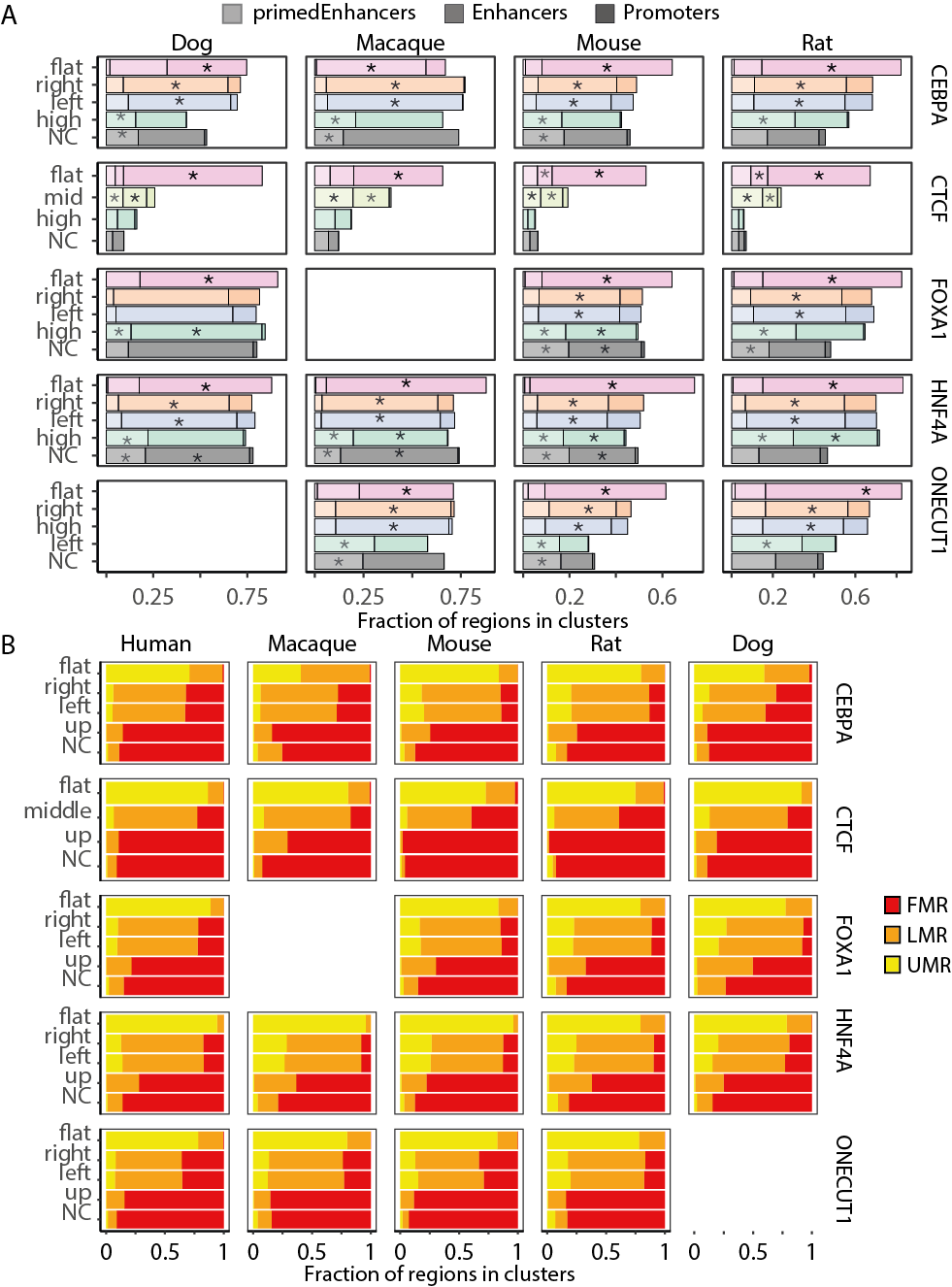
**

**Figure S4: Annotation of TFBRs associated to the clustered 5mC profiles for all species and TFs**. **A)** Proportion of TFBRs that overlap with annotated promoters, active enhancers, and primed enhancers. Asterisks indicate enrichment of annotation type (z-test, p-value < 0.05). **B)** Proportion of TFBRs that overlap with annotated UMRs, LMRs or FMRs. FOXA1 ChIP-seq was not available for macaque, and ONECUT1 was not available for dog.

**
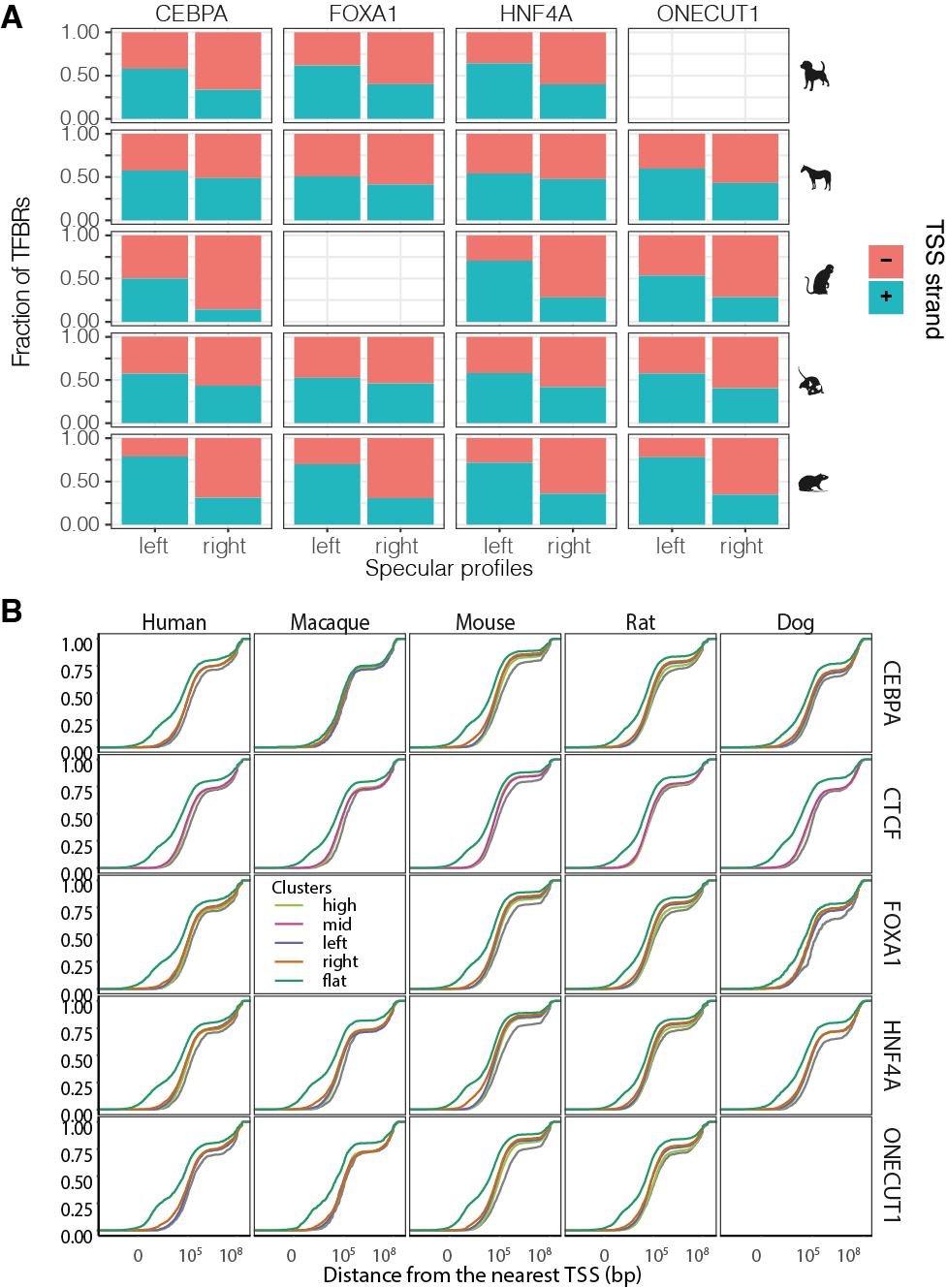
**

**Figure S5**: **A)** Bar plots showing the proportion of TFBRs in the left and right specular clusters that overlap with TSS of genes on the forward (+) or reverse (+) strand. **B)** Cumulative distributions of the distance of each TF binding region from the nearest transcription start site, grouped by 5mC profile. The x axis is in log10 scale. FOXA1 ChIP-seq was not available for macaque, and ONECUT1 was not available for dog.


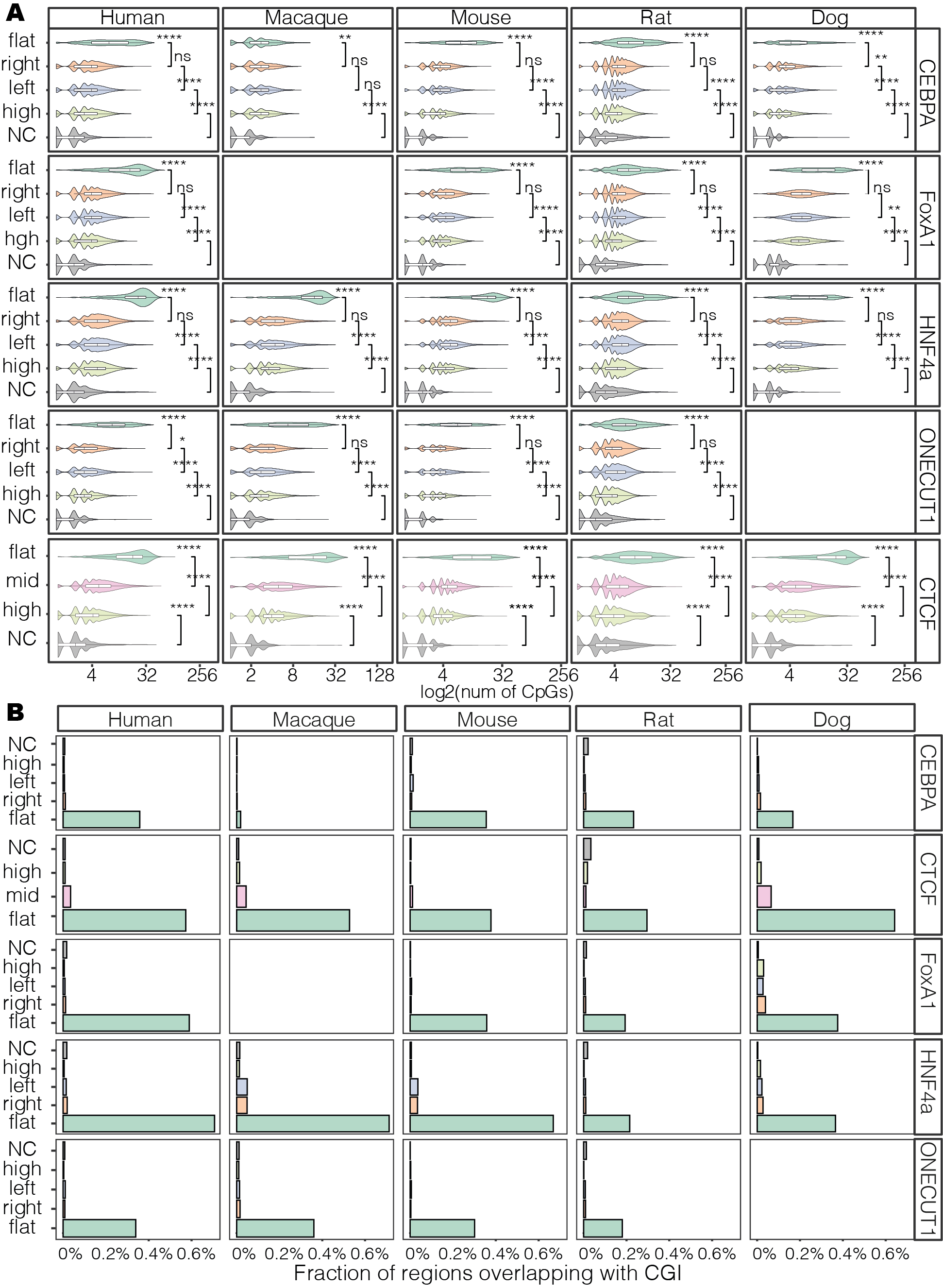


**Figure S6:** **A)** CpG counts distributions at TFBRs associated with different clustered methylation profiles (Wilcoxon Rank test, p-value < 0.05). **B)** Fraction of TFBRs associated with clustered methylation profiles that overlap with CpG islands (CGI). FOXA1 ChIP-seq was not available for macaque, and ONECUT1 was not available for dog.


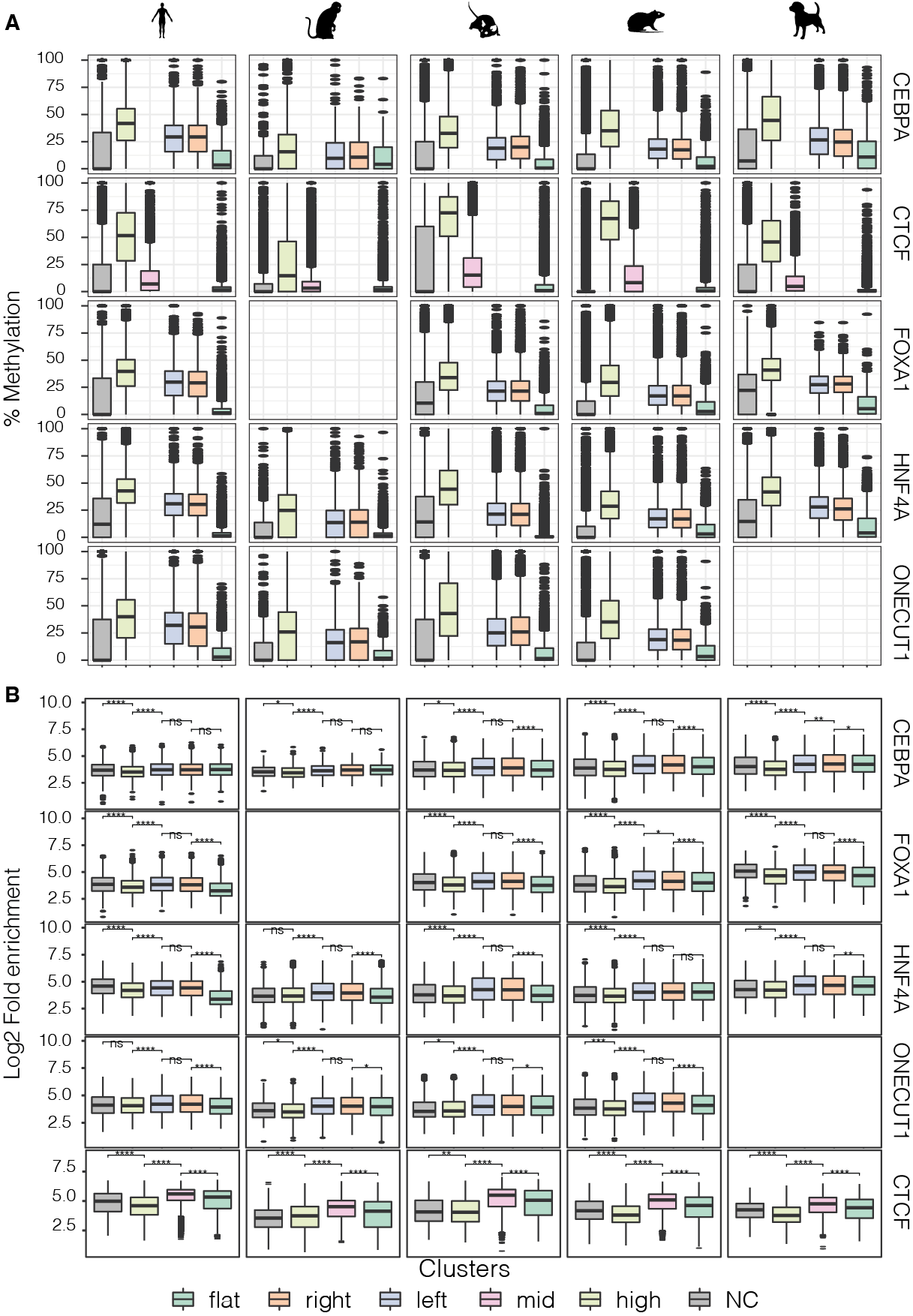


**Figure S7: A)** Average methylation levels distribution of TF binding regions associated with different clustered 5mC profiles. **B)** distribution of fold enrichment values of TF binding regions (ChIP-seq peaks) associated with different clustered methylation profiles (Wilcoxon rank test, ****: p<0.0001, ***: p<0.001, **: p<0.01, *: p<=0.05, ns: p>0.05). FOXA1 ChIP-seq was not available for macaque, and ONECUT1 was not available for dog.


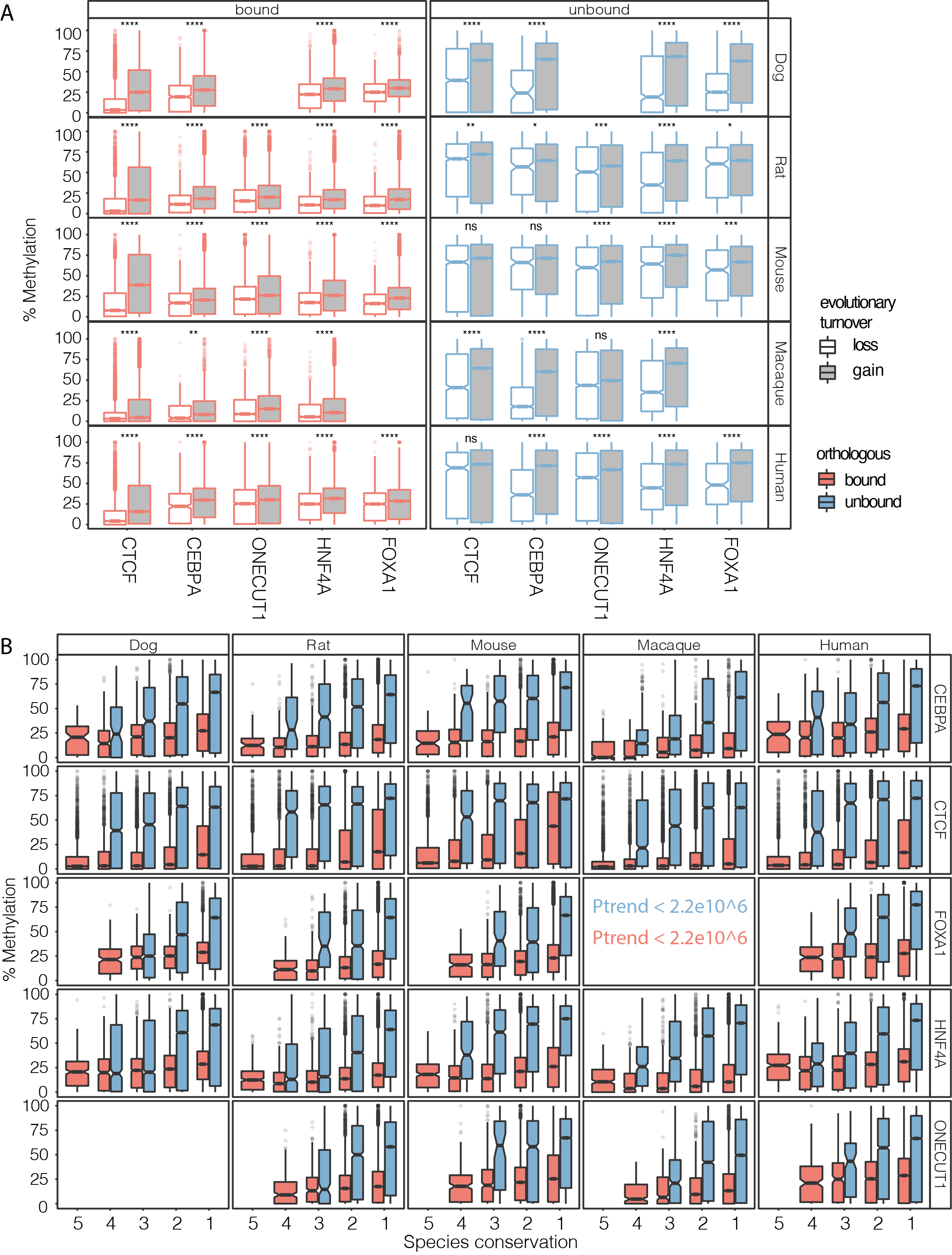


**Figure S8: A)** Average 5mC levels distribution of binding gains sequences compared to binding loss (Wilcoxon rank test, p-value < 0.05; Bonferroni correction). **B)** Average DNA methylation levels distribution of TF bound and unbound regions divided by species conservation categories (Jonckheere-Terpstra trend test, p-values < 2.2e10^6). FOXA1 ChIP-seq was not available for macaque, and ONECUT1 was not available for dog.


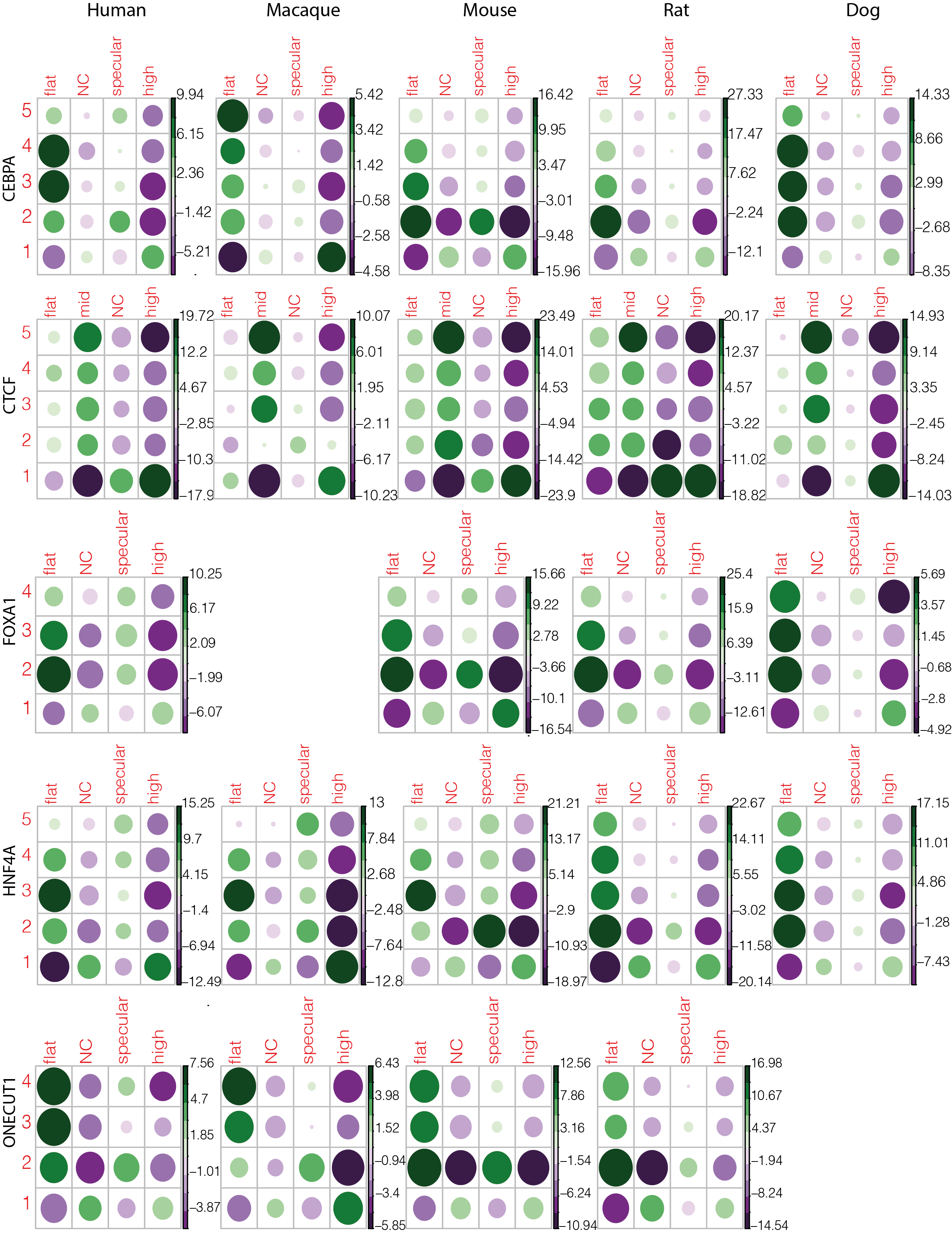


**Figure S9**: Relationships between species conservation and 5mC profiles. Balloon plots show standardized residuals from an association analysis (chi-square test of independence) between 5mC profiles and TF binding conservation categories for dog’s CEBPA and macaque’s CTCF TF binding events. The size of the balloons is proportional to the percentage of contribution to the total Chi-square score .FOXA1 ChIP-seq was not available for macaque, and ONECUT1 was not available for dog.
