# Supplementary material for: DNA methylation patterns of transcription factor binding regions characterize their functional and evolutionary contexts
Source: Genome Biol. 2024 Jun 6;25:146. doi: 10.1186/s13059-024-03218-6 (PMC11155190; doi:10.1186/s13059-024-03218-6)
Supplement: Supplementary file 7 — Additional file 7: Additional File 7.Peer Review History [file 13059_2024_3218_MOESM7_ESM.docx]

**Review history**

**First round of review**

**Reviewer 1**

In this study the authors study DNA methylation patterns in mammals through a comparative approach. The phylogenetic framework employed in this study is the signature of many years of successful genomic research done by the corresponding authors.

The results presented in this paper have the same powerful impact that all TF binding work published previously. How epigenetic modifications (DNA methylation) could affect the evolutionary dynamics of TF binding is the question that the authors address in this paper.

The scientific analyses and results represent a significant advance in the field , I have no specific comments to make here, but in my opinion the figures presenting those results could be, actually have to be, greatly improved. As my postdoc training made me picky about figures, I believe some panels could be rearranged. I would also advise in making the figure legends match the figures as much as possible.

The manuscript could be published as it is, providing authors affine their figure arrangements .
Below are the points I have raised, regardless if they are major or minor, my comments are organized by figure blocks.

Comments

Figure 1

Fig1A : It took me a while to realize that the ChIP-seq signals were shown in "reverse". The color scheme chosen for DNAm clashes with CEBPA and HNF6 colors, which I find confusing. Also the gray alignment blocks (not mentioned in legend btw) are not always well aligned. Those blocks are the first information we see on that figure (the gray is too strong), whereas it should be the low DNAm vs TF binding that should stand up.
I believe that Fig1A could be improved for a better impact.

Fig1C: The blue line at the bottom looks odd to me. Any chance to have a plot without ? Is this conventional ?


Figure 2

Fig2B : I would advise on finer lines for log2(nun of CpGs). The gray blocks appear really unnecessary, and overlap Panel 2B would be improved.
ChIPseq -> ChIP-seq (homogenize with the MS text)
The panel 2D is the most interesting one in that figure, yet it takes a small fraction of the figure. I would advise on improving (squish?) panel 2B, to enlarge panel 2D.

P6L2 : We found that around 80% of binding regions [...[ (Figure 2A). Figure 2A doesn't show that. Should it be Fig2B ? The panel A/B arrangement is confusing to me.

How do you explain the difference of profiles in % methylation between human and macaque ?
Is the TFBSs Dog CEBPA % methylation density profile missing ?


Figure 3

Figure 3A, I would be curious to see the CpG and 5mC profiles at general "enhancers" regions (cCREs from ENCODE, or any other enhancer resource).
For information, the CEBPA slight increase in methylation reminds me of some increase observed in repeat content within CEBPA TFRs.
Graphics and legend for panels C and D need to be seriously improved. It looks like panels have been copy/pasted.
Homogenize line width between panel B (too large) and D.
Personally I would have put for each TF, panel B,C,D horizontally. Like this it would be much easier to understand the links between panel contents. It took me a while to make the connection.
Could you link any methylation profile to genomic locations ? For instance are the Left/Right located near +1/-1 TSS ? Any categories linked with the genomic distance to TSS ?
The "location" results from the Panel C (Active prom etc..) do not really stand up.


Figure 4:

Panel D lacks legend, what does the purple/green scale represent ? What does the size of the balloon represent ?
I find the FigS8A quite interesting, and worth discussing. Any chances in adding a small example of it (ex: just 1TF bound/unbound for 1 species) in Fig4 ?
This would be my "major" point, I am having difficulties following the conclusions on page 11 (L33-L59+) regarding the results presented on panel 4D. I don't know about the other reviewers, but I can't link the textual results and the results shown in figure 4D.

Supp data:

The file EDMscripts.zip I downloaded was empty, so I can't review the "open science" aspect of this work. Any plans for a repo (Github?) with scripts in it, instead of supp zip archive, I believe this would be easier for sharing ?

Thanks for this work.

**Reviewer 2**

The authors generated genome-wide profiles of DNA methylation in livers of five species and compared them to available TF binding sites in those species. They found that DNA methylation levels at TF binding sites have distinct patterns. However, this fits with the existing knowledge of TF binding sites being located in unmethylated regions (UMRs) that are often large CpG dense regions located at CpG island promoters and low methylated regions (LMRs) that are smaller CpG poor regions located at distal enhancers. They further analyze the conservation of DNA methylation levels at TF binding sites and found as expected a coordinated evolution. They also observed that CTCF has distinct patterns which might be due to its specific function.

Data tables 1 & 2 as well as the code used for the analysis was not available for review. This should be provided.

Introduction Page 3 Line 35. I don't think that most CpGs are 0-10% or 70-80% methylated. 70-80% indicated in the reference 16 looked like a mean across all CpGs in the genome. Most CpG methylation goes up to 100%. This is seen in your Figure 1C (and correctly stated in the text) or in reference 16 Figure 1A or reference 30.

Figure 1A. The colors for TF peaks and DNA methylation levels should not overlap, this is misleading. DNA methylation levels are already represented on the y axis, they might not need to be colored.

Page 6 Line 2, 80% of the regions have a CpG in close proximity, please define close proximity (at least in the method).

Motifs logo for the different TFs should be shown in the main figure to see if they may contain CpGs. This could be only one motif from a public database and not all species-specific ones from Figure S1A.

The five TFs chosen for this study do not have prominent CpGs in their motif. Since DNA methylation is more likely to affect TF binding when directly located within its motif, another TF that has a motif containing a CpG should be used for comparison.

Lines in Figure 2B should thinner so we can see all species.

Page 6, "TFBRs have between three and five CpGs". This is represented in log scale in Figure 2B so it would be between 8 and 32 CpGs, please correct. If the TFBRs do not have the same size, the number of CpGs per species is not comparable, this should be normalized at least for the length of the regions but can also be represented as CpG ratio of observed vs expected.

The bimodal distribution observed in Figure 2C is the one described for LMRs (10-50%) and UMRs (10-50%) at TF binding sites. How did you define the limit at 40%? Does it also fit in term of CpG density?

Page 6 "considerable fraction": what is the fraction of CpGs (or regions?) that have intermediated levels?

Figure 2C legend: CTCF does not seem to have a unimodal distribution in the mouse.

Page 6 "hypermethylation occurs more commonly in CEBPA TFBS than in the wider CEBPA TFBR (Figure 2D)": I do not see/understand this point.

Figure 2D line for dog is missing in the CEBPA TFBSs panel.

Figure 2D, is this specific to CEBPA? ONECUT and FOXA1 (Fig S1B) seem to have the same trend.

Page 5, should the title of the section not be "TF binding regions have LOW AND intermediate CpG methylation".

Page 7, how does the title of the section differ from the title of the previous section?

Figure 3A and S2A, B: Plots should all have the same scale for easier comparisons

FigureS2A: For CEBPA in macaque, why is there no enrichment in CpG like for others? What is the quality of the ChIP-seq?

Page 7 "This shows that TFBRs are characterized by key CpGs in close proximity of the binding site which are predominantly unmethylated when the protein is bound.": What are the "key" CpGs?

Figure 3 and S4 do not have the same color scale, legend and order of features, please correct.

Figure 3D and S5: Genomic distances are usually represented in log10 scale (add pseudocount of 1 to see values at 0) with usually a bimodal distribution of the distance to TSS appearing below and above 2Kb. Can you try this scale?

What could explain the strong mirror effect of left/right clusters? There must be something that positions the regions. Could it be genes or other TF binding sites?

Page 10: "orthologous regions that do not bind a TF" should be "that are not bound by a TF"

Figure 4B: Legend says methylation of "bound regions" or "orthologous unbound" but should it be "orthologous bound" like in the figure?

Page 12 last paragraph and page 13 first paragraph: Hypothesis might not hold if analyses were performed on TF which motifs do contain CpGs. The TFs studied might not have CpGs but others binding to the same regions might. TF binding is accepted as a mechanism to initiate and maintain regions unmethylated.

Do other TFs that also have CpGs in their motif have similar patterns than CTCF or is this really CTCF specific?

Your analysis of CTCF binding sites in different species could be compared to a previous one in different cell types: Role of DNA Methylation in Modulating Transcription Factor Occupancy. Maurano MT, et al. Cell Rep. 2015. PMID: 26257180

Do direct binding sites (peaks that have the corresponding motif) have similar methylation patterns as indirect sites (peaks that to not have a motif)?

In the introduction you mentioned that "20-40% of TF binding loss during evolution was not explained by mutations". Then, what is the part explained by DNA methylation? You could take this information into account in your analysis and separate loss and gains according to if they can be explained by mutations or not and then look at methylation levels in those two groups.

**Authors’ response to reviewers**

*We thank the reviewers for their careful reading of our manuscript and their comments. We have revised the manuscript and added a small new results section that directly arose from responding to one of the points below. We believe that we have addressed all the comments made and provided details below in our point-by-point response. We believe that our manuscript has been substantially improved in this process.*

Reviewer reports:

Reviewer #1: In this study the authors study DNA methylation patterns in mammals through a comparative approach. The phylogenetic framework employed in this study is the signature of many years of successful genomic research done by the corresponding authors.

The results presented in this paper have the same powerful impact that all TF binding work published previously. How epigenetic modifications (DNA methylation) could affect the evolutionary dynamics of TF binding is the question that the authors address in this paper.

The scientific analyses and results represent a significant advance in the field , I have no specific comments to make here, but in my opinion the figures presenting those results could be, actually have to be, greatly improved. As my postdoc training made me picky about figures, I believe some panels could be rearranged. I would also advise in making the figure legends match the figures as much as possible.

The manuscript could be published as it is, providing authors affine their figure arrangements.

Below are the points I have raised, regardless if they are major or minor, my comments are organized by figure blocks.

*We thank the reviewer for recognizing the validity of our work and for the thorough examination of our figures. We implemented the reviewer’s suggestions which greatly enhanced the quality of our visualizations.*

Comments

Figure 1

Fig1A : It took me a while to realize that the ChIP-seq signals were shown in "reverse". The color scheme chosen for DNAm clashes with CEBPA and HNF6 colors, which I find confusing. Also the gray alignment blocks (not mentioned in legend btw) are not always well aligned. Those blocks are the first information we see on that figure (the gray is too strong), whereas it should be the low DNAm vs TF binding that should stand up.

I believe that Fig1A could be improved for a better impact.

*We thank the reviewer for their comments which significantly improved Figure 1A. We replotted the ChIP-seq signal tracks so that they are all in the same orientation and removed colours from the DNA methylation tracks altogether. We aligned the alignment blocks more closely and made them light yellow. We now also refer to the alignment blocks in figure legend.*

Fig1C: The blue line at the bottom looks odd to me. Any chance to have a plot without ? Is this conventional ?

*We agree with the reviewer that this was an imperfect visualization. We have removed the blue lines and made the plot axes intersect at (0,0).*

Figure 2

Fig2B : I would advise on finer lines for log2(nun of CpGs). The gray blocks appear really unnecessary, and overlap Panel 2B would be improved.

ChIPseq -> ChIP-seq (homogenize with the MS text)

The panel 2D is the most interesting one in that figure, yet it takes a small fraction of the figure. I would advise on improving (squish?) panel 2B, to enlarge panel 2D.

*We thank the reviewer for the figure enhancement comments. In the process of addressing these comments and similar ones from reviewer 2 about figure content, we conducted additional analysis, which has led to new results and a reorganisation of Figure 2 including a repanelling and moving of one of the previous panels to the supplement. In the revised Figure 2, this reviewer’s comments were addressed as follows:*

-	*Old Figure 2B is now called Supplementary Figure S1B. Density lines are now thinner. We corrected an error in naming the x axis by replacing “log2(num of CpGs)” with “num of CpGs”; the data are not logged, however the x axis is in log scale. The grey areas have been removed.*

-	*“ChIPseq” has been renamed “ChIP-seq” to be consistent across the manuscript and the schematics is now its own labelled subpanel (Figure 2B). In fact, from the reviewer’s later comment we sensed that we did not make it clear that the schematics and the bar plot showing the %TFBSs and %TFBRs were part of the same figure. Therefore, we now separated them, and they each have their own labelled subpanel. This change has been reflected in the figure legend.*

-	*Old Figure 2D has maintained the same panel labe and we believe that in the new version of Figure 2 proportionately takes more space in the figure.*

P6L2 : We found that around 80% of binding regions [...[ (Figure 2A). Figure 2A doesn't show that. Should it be Fig2B ? The panel A/B arrangement is confusing to me.

*As described above, we have rearranged and more effectively labelled the subpanels in Figure 2. Now the ChIP-seq peak schematics is labelled Figure 2B and the bar plot showing the %TFBR and %TFBS containing CpGs is labelled Figure 2S.*

How do you explain the difference of profiles in % methylation between human and macaque

*Macaque’s ChIPseq experiments that were published in Schmidt et al. 2010 (PMID: 20378774) yielded fewer ChIP-seq peaks compared to other species and were noisier than other species’ datasets. This was consistent for all assayed TFs in macaque, but more pronounced for CEBPA. Though the macaque signal is closer to the background frequency (for example, see Figure S2A), the same patterns as those seen for other species’ TFs are still evident. As no result in our manuscript relies exclusively on macaque results, and we no longer have access to the same macaque samples or TF antibodies as published in 2010, we chose to retain the sample. However, we feel that the reviewer has identified a notable difference, and we have now directly addressed the macaque differences in the results.*

Is the TFBSs Dog CEBPA % methylation density profile missing ?

*We apologise that the dog CEBPA profile was missing. The figure was recomputed and it now includes the dog's profile. We thank the reviewer for spotting this.*

Figure 3

Figure 3A, I would be curious to see the CpG and 5mC profiles at general "enhancers" regions (cCREs from ENCODE, or any other enhancer resource).

*The analysis shown in Figure 3A was performed on extended TFBR regions centered around ChIP-seq summits. To compare to orthogonally defined general enhancers, we used H3K27ac ChIP-seq peaks from Roller et al. 2021 (PMID: 33602314) that do not overlap with H3K4me3 peaks and based the analysis on the summit (i.e. center) position of those regions. We then extended -600 and +600 base pairs from the enhancer center and calculated average CpG and 5mC profiles for each genomic position (see figure below). Methylation levels at enhancers are around 75% with a small inflection towards the center, while the CpG profile does not show enrichment at any specific base pair position, although CpG frequency slightly increases concordantly with the methylation decrease. We note that these CpG and methylation patterns are not drastically different from those of random genomic regions (Figure 3A, Supplementary Figure S2) and that further investigation of enhancer methylation profiles would be interesting, but are outside the scope of this manuscript.*

For information, the CEBPA slight increase in methylation reminds me of some increase observed in repeat content within CEBPA TFRs.

*We thank the reviewer for their observation. While addressing points raised by both reviewers, we have now added a new analysis section which specifically looks at the enrichment of hypermethylation in TFs across different groups of transposable elements (Figure 2F). We do indeed now see weak but statistically significant enrichment of hypermethylated CEBPA in certain classes of repeat elements.*

Graphics and legend for panels C and D need to be seriously improved. It looks like panels have been copy/pasted.

Homogenize line width between panel B (too large) and D.

Personally I would have put for each TF, panel B,C,D horizontally. Like this it would be much easier to understand the links between panel contents. It took me a while to make the connection.

*We thank the reviewer for their recommendations which dramatically improved the overall quality of Figure 3C and D. We put panels B, C, D horizontally as suggested, and we homogenized colours and line width across panels. The figure legend was also amended.*

Could you link any methylation profile to genomic locations ? For instance are the Left/Right located near +1/-1 TSS ? Any categories linked with the genomic distance to TSS ?

The "location" results from the Panel C (Active prom etc..) do not really stand up.

*Figure 3D aims to further link the methylation profiles to genomic locations by looking at the distance between the TFBRs in each clustered methylation profile and the closest TSS. After plotting the TFBR-TSS distance in log scale, as reviewer 2 suggested, Figure 3D now better elucidates the tight link between the flat cluster and promoter regions: the cumulative distributions are multimodal and show that TFBRs in the flat cluster are distributed at shorter distances more frequently than the ones assigned to other methylation profiles. In fact, around 30% of the TFBRs belonging to the flat cluster are within 5kb from the closest TSS. TFBRs in the specular, mid and high clusters do not show such distributions and are overall distributed further away from the TSSs.*

*For those Left/Right clusters that do overlap a TSSs, we have now included an analysis of the directionality of transcription in Supplementary Figure S3A and show that the two clusters have a distinct preference to +1 and -1 TSSs.*

Figure 4:

Panel D lacks legend, what does the purple/green scale represent ? What does the size of the balloon represent ?

*We thank the reviewer for raising this point which, together with reviewer 2’s similar comment, gave us the chance to rethink the overall visualization of these results. The colour scale now represents the standardized chi-square residuals (before they were only chi-square residuals): negative residuals indicate a negative association between the methylation profile and the degree of species conservation, while positive residuals indicate a positive association between them. The size of the balloons represents the percentage of each cell’s contribution to the Chi-square score. Both Figure 4D and supplementary Figure S9 now share the same legend across species and TFs. The figure legends have been improved accordingly.*

I find the FigS8A quite interesting, and worth discussing. Any chances in adding a small example of it (ex: just 1TF bound/unbound for 1 species) in Fig4 ?

*We thank the reviewer for this suggestion. Figure 4 was repanelled and now includes an extract of Figure S8 corresponding to Figure 4D (old Figure 4D is now named Figure 4E). We also expanded on the description of this figure in the main text.*

This would be my "major" point, I am having difficulties following the conclusions on page 11 (L33-L59+) regarding the results presented on panel 4D. I don't know about the other reviewers, but I can't link the textual results and the results shown in figure 4D.

*The description of old Figure 4D (now Figure 4E) was entirely rewritten in the main text and the figure legend was expanded to include more details about the statistical test and its interpretation. Briefly, we used the chi-square test of independence to test for the association between the different methylation profiles and the evolutionary conservation categories. In order to perform the test, one needs to calculate the contingency table of all occurrences of methylation profiles and evolutionary conservation. The Chi-square test of independence provides a means to determine whether the two variables are contingent, therefore showing higher frequencies than expected. Figure 4D shows that methylation profiles and evolutionary conservation are indeed related, specifically that flat profiles are associated with higher degree of species conservation but not with lineage-specific binding events, while the opposite was true for the high profiles. Therefore, observed frequencies of flat profiles versus higher conservation and high profile versus lineage-specific binding exceed expectation. We hope that text amendments provide a clearer explanation of our conclusions.*

Supp data:

The file EDMscripts.zip I downloaded was empty, so I can't review the "open science" aspect of this work. Any plans for a repo (Github?) with scripts in it, instead of supp zip archive, I believe this would be easier for sharing ?

*We apologise for the human error which lead to problems with distributing this file. We have created a Github repository with the code:* [*https://github.com/Mavti/evoDNAm_paper*](https://github.com/Mavti/evoDNAm_paper)

Thanks for this work.

Reviewer #2: The authors generated genome-wide profiles of DNA methylation in livers of five species and compared them to available TF binding sites in those species. They found that DNA methylation levels at TF binding sites have distinct patterns. However, this fits with the existing knowledge of TF binding sites being located in unmethylated regions (UMRs) that are often large CpG dense regions located at CpG island promoters and low methylated regions (LMRs) that are smaller CpG poor regions located at distal enhancers. They further analyze the conservation of DNA methylation levels at TF binding sites and found as expected a coordinated evolution. They also observed that CTCF has distinct patterns which might be due to its specific function.

Data tables 1 & 2 as well as the code used for the analysis was not available for review. This should be provided.

*As mentioned in the response to reviewer 1, we apologize for accidentally distributing an empty script file and not including two data tables. This was both due to human error and is in contrast to years of our commitment to openly sharing our results. We have now uploaded the scripts to github (*[*https://github.com/Mavti/evoDNAm_paper*](https://github.com/Mavti/evoDNAm_paper)*) and made sure all supplementary materials are uploaded.*

Introduction Page 3 Line 35. I don't think that most CpGs are 0-10% or 70-80% methylated. 70-80% indicated in the reference 16 looked like a mean across all CpGs in the genome. Most CpG methylation goes up to 100%. This is seen in your Figure 1C (and correctly stated in the text) or in reference 16 Figure 1A or reference 30.

*We thank the reviewer for this comment, we indeed erroneously wrote 70-80% when in reality meant “70-100%”. This is now correctly stated in the text.*

Figure 1A. The colors for TF peaks and DNA methylation levels should not overlap, this is misleading. DNA methylation levels are already represented on the y axis, they might not need to be colored.

*We thank the reviewer for the figure enhancement comments. In the process of addressing this and reviewer #1’s comments, we greatly improved the quality of Figure 1. We removed the colour scale on the DNA methylation track and we flipped the ChIP-seq tracks. We also made the alignment blocks lighter*.

Page 6 Line 2, 80% of the regions have a CpG in close proximity, please define close proximity (at least in the method).

*By “close proximity of the binding site” we meant “within the area of the TFBRs” which depends on the average length of peaks within each species and TF dataset, but generally corresponds to around 250bp. We amended the main text to make this point clearer.*

Motifs logo for the different TFs should be shown in the main figure to see if they may contain CpGs. This could be only one motif from a public database and not all species-specific ones from Figure S1A.

*We agree that the motif logo should appear in the main figure and they are now part of Figure 2A. As suggested by the reviewer, we chose to show only one motif - the one we de novo extracted from the human ChIP-seq samples. This is further appropriate to be representative of all species due to the known lack of TF motif divergence between mammals as shown in Schmidt et al. 2010 (PMID: 20378774).*

The five TFs chosen for this study do not have prominent CpGs in their motif. Since DNA methylation is more likely to affect TF binding when directly located within its motif, another TF that has a motif containing a CpG should be used for comparison.

*We have now included the typical motif logos in Figure 2A, and they show that high-scoring CpG sequences are present in the position weight matrices of CTCF and CEBPA at positions 15 and 7, respectively. Furthermore, CEBPA has alternative specificity for methylated sequences (PMID: 21059933****)****, while CTCF may be inhibited by 5mC in specific contexts, while still binding methylated sequences upon participating in cooperating binding (PMID: 26257180****)****.*

*We agree with this reviewer that the TFs used in this study do not show a prominent CpG in their motif when binding in liver, and that the results may not be fully generalizable to the whole spectrum of TF binding. For this reason, we tone down some of the conclusions in the manuscript.*

Lines in Figure 2B should thinner so we can see all species.

*We reduced the line width in old Figure 2B. In response to other reviewer comments it was also moved to the supplement and renamed Figure S1B.*

Page 6, "TFBRs have between three and five CpGs". This is represented in log scale in Figure 2B so it would be between 8 and 32 CpGs, please correct. If the TFBRs do not have the same size, the number of CpGs per species is not comparable, this should be normalized at least for the length of the regions but can also be represented as CpG ratio of observed vs expected.

*We apologise for erroneously writing “log2(num of CpGs)” in the x axis title of Figure 2B: the data were not logged, but the axis was in log2 scale. We corrected the x axis title as “num of CpGs”. As we did not intend to perform a direct comparison between species in Figure 2, nor compare within the text, we limited the normalization of the TFBRs only within each experiment.*

The bimodal distribution observed in Figure 2C is the one described for LMRs (10-50%) and UMRs (10-50%) at TF binding sites. How did you define the limit at 40%? Does it also fit in term of CpG density?

*To clarify this point, we now report the modes extracted for each distribution in Supplementary Table 2 and described the process in the Method section. Figure 3 and corresponding Supplementary Figures S4, S6, & S7 further investigate the intermediate methylation pattern, which is linked to the specular profiles. Supplementary Figure S6 shows the distribution of the number of CpGs within TFBRs classified with each methylation profile and shows that specular profiles - largely corresponding to LMRs - have intermediate CpG densities with higher amount of CpGs than High profile TFBRs (largely in FMRs), but less than the Flat profile TFBRs (mainly in UMRs).*

Page 6 "considerable fraction": what is the fraction of CpGs (or regions?) that have intermediated levels?

*We now report the fraction of TFBRs that have average methylation within an interval defined as the highest mode +/- 15 in Supplementary Table 2.*

Figure 2C legend: CTCF does not seem to have a unimodal distribution in the mouse.

*We deeply thank the reviewer for this insightful comment which led us to generate new results. CTCF distributions are indeed not unimodal. This is confirmed by the excess mass test we performed on each species' distribution. In fact, all species’ CTCF distributions are bimodal, not only the one in mouse. This is more evident when visualising the average methylation distribution in log scale. These results show that the whole rodent clade had higher densities of hypermethylation values; indeed, mouse and rat have distributions with hypermethylated modes around 80% (Supplementary Table 2 shows all modes). We next explored if these hypomethylated regions arose from recent transposable element expansions which are known to have shaped CTCF regulatory networks in mammals. Our results show that the rodents indeed have a higher fraction of hypermethylated TFBRs overlapping TEs than other species. To accommodate these newer results, Figure 2 has been repanelled and the main text and method section expanded.*

Page 6 "hypermethylation occurs more commonly in CEBPA TFBS than in the wider CEBPA TFBR (Figure 2D)": I do not see/understand this point.

*Figure 2D shows higher densities of hypermethylated values in CEBPA’s TFBS distributions that do not appear for TFBRs. This indicates that a subpopulation of CEBPA TFBS have CpGs that are hypermethylated and this differs from the remaining TF’s distributions. To better elucidate this point, we generated a new figure panel named Supplementary Figure S1D where we show that the fraction of motifs with methylation above 75% exceeds the fraction of TFBRs with methylation above 75% only for CEBPA.*

Figure 2D line for dog is missing in the CEBPA TFBSs panel.

*We thank the reviewer for spotting this. Dog’s CEBPA line was indeed missing and a new Figure 2D has been generated.*

Figure 2D, is this specific to CEBPA? ONECUT and FOXA1 (Fig S1B) seem to have the same trend.

*It is indeed a general trend that the individual CpGs within the TFBRs and TFBSs have intermediate methylation levels but we selected CEBPA as a representative of this pattern. However, CEBPA is the only TF showing higher density of hypermethylation values within TFBSs as opposed to TFBRs.*

Page 5, should the title of the section not be "TF binding regions have LOW AND intermediate CpG methylation".

*The section title has been amended.*

Page 7, how does the title of the section differ from the title of the previous section?

*In the previous paragraph we meant to emphasise that TFs bind at DNA sequences with different methylation patterns which we were able to characterise, while in the next section we meant to highlight that these alternative methylation patterns correspond to different functional genomic contexts. We amended the paragraph titles to better reflect this.*

Figure 3A and S2A, B: Plots should all have the same scale for easier comparisons.

*We thank the reviewer for this suggestion, Figure 3A and S2A,B now all have the same scale.*

FigureS2A: For CEBPA in macaque, why is there no enrichment in CpG like for others? What is the quality of the ChIP-seq?

*As we mentioned in an earlier response to reviewer 1 and now explicitly address in the Result text, macaque’s CEBPA ChIP-seq data are indeed noisier than other TFs. However, the same trends are still present, and no specific result rests on the macaque ChIP-seq alonoe.*

Page 7 "This shows that TFBRs are characterized by key CpGs in close proximity of the binding site which are predominantly unmethylated when the protein is bound.": What are the "key" CpGs?

*As the CpG frequency profiles show increased CpG frequency in the immediate surrounding of the binding site despite the absence of prominent CpGs within the motif logos, we conclude that these CpGs must be important for the successful binding of the TF to their target sequence (hence, “key”). However, we agree that these “key” CpGs have not been further characterised so we removed the word “key” from the text.*

Figure 3 and S4 do not have the same color scale, legend and order of features, please correct.

*Figure 3 and S4 have been homogenized accordingly. Thank you.*

Figure 3D and S5: Genomic distances are usually represented in log10 scale (add pseudocount of 1 to see values at 0) with usually a bimodal distribution of the distance to TSS appearing below and above 2Kb. Can you try this scale?

*We thank the reviewer for this suggestion. Figure 3D is now in log10 scale. Indeed, the multimodal distribution appears, but it differs between TFBRs with distinct methylation profiles. We can now show more clearly that TFBRs in the flat clusters - which overlap active promoter regions - are more frequently distributed at shorter distances to TSSs. TFBRs in the remaining clusters have a mode at longer distances.*

What could explain the strong mirror effect of left/right clusters? There must be something that positions the regions. Could it be genes or other TF binding sites?

*We agree with the review that the strong mirror effect is an interesting property of these clusters. We further explored whether it can be explained with the directionality of transcription by overlapping the TFBRs with annotated TSSs and examining their direction of transcription. In Supplementary Figure S5A we now show that TFBRs with left profiles are more frequently located overlapping TSSs on the forward strand, while TFBRs with right profiles overlap those on the reverse strand, indicating that the lowly methylated regions of the profiles correspond to the first exon which is typically unmethylated.*

Page 10: "orthologous regions that do not bind a TF" should be "that are not bound by a TF"

*Thank you. It has been corrected accordingly.*

Figure 4B: Legend says methylation of "bound regions" or "orthologous unbound" but should it be "orthologous bound" like in the figure?

*Thank you. It has been corrected accordingly.*

Page 12 last paragraph and page 13 first paragraph: Hypothesis might not hold if analyses were performed on TF which motifs do contain CpGs. The TFs studied might not have CpGs but others binding to the same regions might. TF binding is accepted as a mechanism to initiate and maintain regions unmethylated.

*We agree with this comment and have now clarified in the text that the hypothesis might only hold for the TFs included in our study, but alternative mechanisms for TF with a direct interaction with methylated CpGs might exist.*

Do other TFs that also have CpGs in their motif have similar patterns than CTCF or is this really CTCF specific?

*We agree with this insightful comment and suggest in the main text that the analyses of different TFs with specificity rules more similar to CTCF might clarify whether the patterns that we see are ultimately CTCF specific.*

Your analysis of CTCF binding sites in different species could be compared to a previous one in different cell types: Role of DNA Methylation in Modulating Transcription Factor Occupancy. Maurano MT, et al. Cell Rep. 2015. PMID: 26257180

*We thank the reviewer for pointing out a relevant publication, and have used it to expand on the relevance of CTCF’s context effects and conservation in the Discussion. We use it to draw parallels between alterations of the DNA methylation landscape between different cell types and between species.*

Do direct binding sites (peaks that have the corresponding motif) have similar methylation patterns as indirect sites (peaks that to not have a motif)?

*Thank you for this interesting question. As shown below, there is not a striking difference between TFBRs with a discoverable motif within its region (“+ motif”) and TFBRs without (“- motif”). Both average methylation distributions (here in log scale) are bimodal, however, TFBRs without an annotated motif have higher densities both of unmethylated values and intermediately methylated values. We believe that these differences are merely due to the fact TFBRs with annotated motifs are a minority of all the TFBRs as our de novo motif identification strategy was indeed relatively stringent.*

In the introduction you mentioned that "20-40% of TF binding loss during evolution was not explained by mutations". Then, what is the part explained by DNA methylation? You could take this information into account in your analysis and separate loss and gains according to if they can be explained by mutations or not and then look at methylation levels in those two groups.

*This is an extremely important point that we have considered along the way, but did not pursue due to insufficient sample size to make statistically robust conclusions about the effect of DNA methylation on TF binding loss. For this analysis, we would need CpGs at the binding site of TFBRs which were evolutionarily lost. Firstly, less than 10% of TF binding motifs contain at least one CpG which could be methylated (Fig 2A). This set is even smaller if we consider that the motif would need to be completely conserved, including the CpG site, to unequivocally assess only the effect of methylation on binding divergence. Finally, there is an inherent bias in the distribution of CpG-motifs within different genomic contexts. A larger fraction is located within CpG denser regions - like promoters - rather than other CpG poorer contexts (figure below). Promoters are the most evolutionarily conserved genomic contexts, are consistently unmethylated and functionally conserved.*

**Second round of review**

**Reviewer 2**

I thank the authors for their answers, changes in the manuscript and figures and new analyses. However, whereas CTCF may have few motif occurrences with a CpG on position 15, CEBPA does not. The GC in position 7 is not a CpG and will not get methylated. There is therefore no prominent CpGs in the motifs of the TFs studied in this study which strongly limits conclusions about the impact of DNA methylation on TF binding and evolution.
The authors further agree that studying the effect of mutations on TF binding loss is an important question which cannot be answered without analyzing TFs that have CpGs in their motifs.

**Authors’ response to reviewers**

Reviewer #2: I thank the authors for their answers, changes in the manuscript and figures and new analyses. However, whereas CTCF may have few motif occurrences with a CpG on position 15, CEBPA does not. The GC in position 7 is not a CpG and will not get methylated. There is therefore no prominent CpGs in the motifs of the TFs studied in this study which strongly limits conclusions about the impact of DNA methylation on TF binding and evolution.

*We apologise for the typo in our response stating that CpG is in position 7 of the CEBPA motif. This should be position 6 and there are between 7-11% of CEBPA binding sites (depending on species) in our data that have CpGs and could be methylated. We have updated the results section on page 6 to report the fraction of binding sites with CpGs in their motifs and to make the point that CpGs are generally more common in the sequence surrounding the motif – which we refer to as binding regions – than in the motif itself.*

*We believe we have been careful in the conclusions that we have drawn from this pattern of CpGs in the regions and the motifs and do not believe that these are “strongly limited”. First, although there are relatively few bound motifs with CpGs, the majority of binding regions have CpGs (Figure 2A) which we state may influence cooperative binding partners based on previous observations that TF binding changes when binding partners are disrupted. More importantly, we show that there are significant differences in methylation levels between bound and unbound orthologous regions across species (Figure 4B, 4C, 4D). We argue that the results in Figure 4 strongly suggest that at least part of the story for why transcription factor binding changes rapidly across species, even when the core motif does not change, is DNA methylation.*

The authors further agree that studying the effect of mutations on TF binding loss is an important question which cannot be answered without analyzing TFs that have CpGs in their motifs.

*We believe this comment is a misunderstanding of our response to the very last comment by reviewer 2 in the original response below. Specifically, the reviewer asked what part of the binding loss in evolution that is not explained by mutations can be explained by DNA methylation. The reviewer then proposed an analysis of separating gains and losses according to whether or not they can be explained by mutations and then comparing the methylation in those groups. We responded to this suggestion of analysis by stating that there are too few cases to achieve statistical significance based on 20-40% binding loss without obvious sequence mutation and 3-18% of binding motifs with CpGs.*

*We absolutely do not agree that it is impossible to address the question of whether change in DNA methylation is associated with loss of TF binding. Indeed, we have done so in Figure 4 using the wider binding region and see both an overall association (Figure 4B) and an effect following binding conservation that is similar to what is seen for other biological assays when compared to binding conservation (Figure 4C).*

*We have added some additional information to the relevant results section on page 12 and an additional sentence to the discussion on page 15 to make these points clearer and clarify the limits of our conclusions.*

*------	Everything following is our previous response to reviewers. -------*

*We thank the reviewers for their careful reading of our manuscript and their comments. We have revised the manuscript and added a small new results section that directly arose from responding to one of the points below. We believe that we have addressed all the comments made and provided details below in our point-by-point response. We believe that our manuscript has been substantially improved in this process.*

Reviewer reports:

Reviewer #1: In this study the authors study DNA methylation patterns in mammals through a comparative approach. The phylogenetic framework employed in this study is the signature of many years of successful genomic research done by the corresponding authors.

The results presented in this paper have the same powerful impact that all TF binding work published previously. How epigenetic modifications (DNA methylation) could affect the evolutionary dynamics of TF binding is the question that the authors address in this paper.

The scientific analyses and results represent a significant advance in the field , I have no specific comments to make here, but in my opinion the figures presenting those results could be, actually have to be, greatly improved. As my postdoc training made me picky about figures, I believe some panels could be rearranged. I would also advise in making the figure legends match the figures as much as possible.

The manuscript could be published as it is, providing authors affine their figure arrangements.

Below are the points I have raised, regardless if they are major or minor, my comments are organized by figure blocks.

*We thank the reviewer for recognizing the validity of our work and for the thorough examination of our figures. We implemented the reviewer’s suggestions which greatly enhanced the quality of our visualizations.*

Comments

Figure 1

Fig1A : It took me a while to realize that the ChIP-seq signals were shown in "reverse". The color scheme chosen for DNAm clashes with CEBPA and HNF6 colors, which I find confusing. Also the gray alignment blocks (not mentioned in legend btw) are not always well aligned. Those blocks are the first information we see on that figure (the gray is too strong), whereas it should be the low DNAm vs TF binding that should stand up.

I believe that Fig1A could be improved for a better impact.

*We thank the reviewer for their comments which significantly improved Figure 1A. We replotted the ChIP-seq signal tracks so that they are all in the same orientation and removed colours from the DNA methylation tracks altogether. We aligned the alignment blocks more closely and made them light yellow. We now also refer to the alignment blocks in figure legend.*

Fig1C: The blue line at the bottom looks odd to me. Any chance to have a plot without ? Is this conventional ?

*We agree with the reviewer that this was an imperfect visualization. We have removed the blue lines and made the plot axes intersect at (0,0).*

Figure 2

Fig2B : I would advise on finer lines for log2(nun of CpGs). The gray blocks appear really unnecessary, and overlap Panel 2B would be improved.

ChIPseq -> ChIP-seq (homogenize with the MS text)

The panel 2D is the most interesting one in that figure, yet it takes a small fraction of the figure. I would advise on improving (squish?) panel 2B, to enlarge panel 2D.

*We thank the reviewer for the figure enhancement comments. In the process of addressing these comments and similar ones from reviewer 2 about figure content, we conducted additional analysis, which has led to new results and a reorganisation of Figure 2 including a repanelling and moving of one of the previous panels to the supplement. In the revised Figure 2, this reviewer’s comments were addressed as follows:*

-	*Old Figure 2B is now called Supplementary Figure S1B. Density lines are now thinner. We corrected an error in naming the x axis by replacing “log2(num of CpGs)” with “num of CpGs”; the data are not logged, however the x axis is in log scale. The grey areas have been removed.*

-	*“ChIPseq” has been renamed “ChIP-seq” to be consistent across the manuscript and the schematics is now its own labelled subpanel (Figure 2B). In fact, from the reviewer’s later comment we sensed that we did not make it clear that the schematics and the bar plot showing the %TFBSs and %TFBRs were part of the same figure. Therefore, we now separated them, and they each have their own labelled subpanel. This change has been reflected in the figure legend.*

-	*Old Figure 2D has maintained the same panel labe and we believe that in the new version of Figure 2 proportionately takes more space in the figure.*

P6L2 : We found that around 80% of binding regions [...[ (Figure 2A). Figure 2A doesn't show that. Should it be Fig2B ? The panel A/B arrangement is confusing to me.

*As described above, we have rearranged and more effectively labelled the subpanels in Figure 2. Now the ChIP-seq peak schematics is labelled Figure 2B and the bar plot showing the %TFBR and %TFBS containing CpGs is labelled Figure 2S.*

How do you explain the difference of profiles in % methylation between human and macaque

*Macaque’s ChIPseq experiments that were published in Schmidt et al. 2010 (PMID: 20378774) yielded fewer ChIP-seq peaks compared to other species and were noisier than other species’ datasets. This was consistent for all assayed TFs in macaque, but more pronounced for CEBPA. Though the macaque signal is closer to the background frequency (for example, see Figure S2A), the same patterns as those seen for other species’ TFs are still evident. As no result in our manuscript relies exclusively on macaque results, and we no longer have access to the same macaque samples or TF antibodies as published in 2010, we chose to retain the sample. However, we feel that the reviewer has identified a notable difference, and we have now directly addressed the macaque differences in the results.*

Is the TFBSs Dog CEBPA % methylation density profile missing ?

*We apologise that the dog CEBPA profile was missing. The figure was recomputed and it now includes the dog's profile. We thank the reviewer for spotting this.*

Figure 3

Figure 3A, I would be curious to see the CpG and 5mC profiles at general "enhancers" regions (cCREs from ENCODE, or any other enhancer resource).

*The analysis shown in Figure 3A was performed on extended TFBR regions centered around ChIP-seq summits. To compare to orthogonally defined general enhancers, we used H3K27ac ChIP-seq peaks from Roller et al. 2021 (PMID: 33602314) that do not overlap with H3K4me3 peaks and based the analysis on the summit (i.e. center) position of those regions. We then extended -600 and +600 base pairs from the enhancer center and calculated average CpG and 5mC profiles for each genomic position (see figure below). Methylation levels at enhancers are around 75% with a small inflection towards the center, while the CpG profile does not show enrichment at any specific base pair position, although CpG frequency slightly increases concordantly with the methylation decrease. We note that these CpG and methylation patterns are not drastically different from those of random genomic regions (Figure 3A, Supplementary Figure S2) and that further investigation of enhancer methylation profiles would be interesting, but are outside the scope of this manuscript.*

For information, the CEBPA slight increase in methylation reminds me of some increase observed in repeat content within CEBPA TFRs.

*We thank the reviewer for their observation. While addressing points raised by both reviewers, we have now added a new analysis section which specifically looks at the enrichment of hypermethylation in TFs across different groups of transposable elements (Figure 2F). We do indeed now see weak but statistically significant enrichment of hypermethylated CEBPA in certain classes of repeat elements.*

Graphics and legend for panels C and D need to be seriously improved. It looks like panels have been copy/pasted.

Homogenize line width between panel B (too large) and D.

Personally I would have put for each TF, panel B,C,D horizontally. Like this it would be much easier to understand the links between panel contents. It took me a while to make the connection.

*We thank the reviewer for their recommendations which dramatically improved the overall quality of Figure 3C and D. We put panels B, C, D horizontally as suggested, and we homogenized colours and line width across panels. The figure legend was also amended.*

Could you link any methylation profile to genomic locations ? For instance are the Left/Right located near +1/-1 TSS ? Any categories linked with the genomic distance to TSS ?

The "location" results from the Panel C (Active prom etc..) do not really stand up.

*Figure 3D aims to further link the methylation profiles to genomic locations by looking at the distance between the TFBRs in each clustered methylation profile and the closest TSS. After plotting the TFBR-TSS distance in log scale, as reviewer 2 suggested, Figure 3D now better elucidates the tight link between the flat cluster and promoter regions: the cumulative distributions are multimodal and show that TFBRs in the flat cluster are distributed at shorter distances more frequently than the ones assigned to other methylation profiles. In fact, around 30% of the TFBRs belonging to the flat cluster are within 5kb from the closest TSS. TFBRs in the specular, mid and high clusters do not show such distributions and are overall distributed further away from the TSSs.*

*For those Left/Right clusters that do overlap a TSSs, we have now included an analysis of the directionality of transcription in Supplementary Figure S3A and show that the two clusters have a distinct preference to +1 and -1 TSSs.*

Figure 4:

Panel D lacks legend, what does the purple/green scale represent ? What does the size of the balloon represent ?

*We thank the reviewer for raising this point which, together with reviewer 2’s similar comment, gave us the chance to rethink the overall visualization of these results. The colour scale now represents the standardized chi-square residuals (before they were only chi-square residuals): negative residuals indicate a negative association between the methylation profile and the degree of species conservation, while positive residuals indicate a positive association between them. The size of the balloons represents the percentage of each cell’s contribution to the Chi-square score. Both Figure 4D and supplementary Figure S9 now share the same legend across species and TFs. The figure legends have been improved accordingly.*

I find the FigS8A quite interesting, and worth discussing. Any chances in adding a small example of it (ex: just 1TF bound/unbound for 1 species) in Fig4 ?

*We thank the reviewer for this suggestion. Figure 4 was repanelled and now includes an extract of Figure S8 corresponding to Figure 4D (old Figure 4D is now named Figure 4E). We also expanded on the description of this figure in the main text.*

This would be my "major" point, I am having difficulties following the conclusions on page 11 (L33-L59+) regarding the results presented on panel 4D. I don't know about the other reviewers, but I can't link the textual results and the results shown in figure 4D.

*The description of old Figure 4D (now Figure 4E) was entirely rewritten in the main text and the figure legend was expanded to include more details about the statistical test and its interpretation. Briefly, we used the chi-square test of independence to test for the association between the different methylation profiles and the evolutionary conservation categories. In order to perform the test, one needs to calculate the contingency table of all occurrences of methylation profiles and evolutionary conservation. The Chi-square test of independence provides a means to determine whether the two variables are contingent, therefore showing higher frequencies than expected. Figure 4D shows that methylation profiles and evolutionary conservation are indeed related, specifically that flat profiles are associated with higher degree of species conservation but not with lineage-specific binding events, while the opposite was true for the high profiles. Therefore, observed frequencies of flat profiles versus higher conservation and high profile versus lineage-specific binding exceed expectation. We hope that text amendments provide a clearer explanation of our conclusions.*

Supp data:

The file EDMscripts.zip I downloaded was empty, so I can't review the "open science" aspect of this work. Any plans for a repo (Github?) with scripts in it, instead of supp zip archive, I believe this would be easier for sharing ?

*We apologise for the human error which lead to problems with distributing this file. We have created a Github repository with the code:* [*https://github.com/Mavti/evoDNAm_paper*](https://github.com/Mavti/evoDNAm_paper)

Thanks for this work.

Reviewer #2: The authors generated genome-wide profiles of DNA methylation in livers of five species and compared them to available TF binding sites in those species. They found that DNA methylation levels at TF binding sites have distinct patterns. However, this fits with the existing knowledge of TF binding sites being located in unmethylated regions (UMRs) that are often large CpG dense regions located at CpG island promoters and low methylated regions (LMRs) that are smaller CpG poor regions located at distal enhancers. They further analyze the conservation of DNA methylation levels at TF binding sites and found as expected a coordinated evolution. They also observed that CTCF has distinct patterns which might be due to its specific function.

Data tables 1 & 2 as well as the code used for the analysis was not available for review. This should be provided.

*As mentioned in the response to reviewer 1, we apologize for accidentally distributing an empty script file and not including two data tables. This was both due to human error and is in contrast to years of our commitment to openly sharing our results. We have now uploaded the scripts to github (*[*https://github.com/Mavti/evoDNAm_paper*](https://github.com/Mavti/evoDNAm_paper)*) and made sure all supplementary materials are uploaded.*

Introduction Page 3 Line 35. I don't think that most CpGs are 0-10% or 70-80% methylated. 70-80% indicated in the reference 16 looked like a mean across all CpGs in the genome. Most CpG methylation goes up to 100%. This is seen in your Figure 1C (and correctly stated in the text) or in reference 16 Figure 1A or reference 30.

*We thank the reviewer for this comment, we indeed erroneously wrote 70-80% when in reality meant “70-100%”. This is now correctly stated in the text.*

Figure 1A. The colors for TF peaks and DNA methylation levels should not overlap, this is misleading. DNA methylation levels are already represented on the y axis, they might not need to be colored.

*We thank the reviewer for the figure enhancement comments. In the process of addressing this and reviewer #1’s comments, we greatly improved the quality of Figure 1. We removed the colour scale on the DNA methylation track and we flipped the ChIP-seq tracks. We also made the alignment blocks lighter*.

Page 6 Line 2, 80% of the regions have a CpG in close proximity, please define close proximity (at least in the method).

*By “close proximity of the binding site” we meant “within the area of the TFBRs” which depends on the average length of peaks within each species and TF dataset, but generally corresponds to around 250bp. We amended the main text to make this point clearer.*

Motifs logo for the different TFs should be shown in the main figure to see if they may contain CpGs. This could be only one motif from a public database and not all species-specific ones from Figure S1A.

*We agree that the motif logo should appear in the main figure and they are now part of Figure 2A. As suggested by the reviewer, we chose to show only one motif - the one we de novo extracted from the human ChIP-seq samples. This is further appropriate to be representative of all species due to the known lack of TF motif divergence between mammals as shown in Schmidt et al. 2010 (PMID: 20378774).*

The five TFs chosen for this study do not have prominent CpGs in their motif. Since DNA methylation is more likely to affect TF binding when directly located within its motif, another TF that has a motif containing a CpG should be used for comparison.

*We have now included the typical motif logos in Figure 2A, and they show that high-scoring CpG sequences are present in the position weight matrices of CTCF and CEBPA at positions 15 and 7, respectively. Furthermore, CEBPA has alternative specificity for methylated sequences (PMID: 21059933****)****, while CTCF may be inhibited by 5mC in specific contexts, while still binding methylated sequences upon participating in cooperating binding (PMID: 26257180****)****.*

*We agree with this reviewer that the TFs used in this study do not show a prominent CpG in their motif when binding in liver, and that the results may not be fully generalizable to the whole spectrum of TF binding. For this reason, we tone down some of the conclusions in the manuscript.*

Lines in Figure 2B should thinner so we can see all species.

*We reduced the line width in old Figure 2B. In response to other reviewer comments it was also moved to the supplement and renamed Figure S1B.*

Page 6, "TFBRs have between three and five CpGs". This is represented in log scale in Figure 2B so it would be between 8 and 32 CpGs, please correct. If the TFBRs do not have the same size, the number of CpGs per species is not comparable, this should be normalized at least for the length of the regions but can also be represented as CpG ratio of observed vs expected.

*We apologise for erroneously writing “log2(num of CpGs)” in the x axis title of Figure 2B: the data were not logged, but the axis was in log2 scale. We corrected the x axis title as “num of CpGs”. As we did not intend to perform a direct comparison between species in Figure 2, nor compare within the text, we limited the normalization of the TFBRs only within each experiment.*

The bimodal distribution observed in Figure 2C is the one described for LMRs (10-50%) and UMRs (10-50%) at TF binding sites. How did you define the limit at 40%? Does it also fit in term of CpG density?

*To clarify this point, we now report the modes extracted for each distribution in Supplementary Table 2 and described the process in the Method section. Figure 3 and corresponding Supplementary Figures S4, S6, & S7 further investigate the intermediate methylation pattern, which is linked to the specular profiles. Supplementary Figure S6 shows the distribution of the number of CpGs within TFBRs classified with each methylation profile and shows that specular profiles - largely corresponding to LMRs - have intermediate CpG densities with higher amount of CpGs than High profile TFBRs (largely in FMRs), but less than the Flat profile TFBRs (mainly in UMRs).*

Page 6 "considerable fraction": what is the fraction of CpGs (or regions?) that have intermediated levels?

*We now report the fraction of TFBRs that have average methylation within an interval defined as the highest mode +/- 15 in Supplementary Table 2.*

Figure 2C legend: CTCF does not seem to have a unimodal distribution in the mouse.

*We deeply thank the reviewer for this insightful comment which led us to generate new results. CTCF distributions are indeed not unimodal. This is confirmed by the excess mass test we performed on each species' distribution. In fact, all species’ CTCF distributions are bimodal, not only the one in mouse. This is more evident when visualising the average methylation distribution in log scale. These results show that the whole rodent clade had higher densities of hypermethylation values; indeed, mouse and rat have distributions with hypermethylated modes around 80% (Supplementary Table 2 shows all modes). We next explored if these hypomethylated regions arose from recent transposable element expansions which are known to have shaped CTCF regulatory networks in mammals. Our results show that the rodents indeed have a higher fraction of hypermethylated TFBRs overlapping TEs than other species. To accommodate these newer results, Figure 2 has been repanelled and the main text and method section expanded.*

Page 6 "hypermethylation occurs more commonly in CEBPA TFBS than in the wider CEBPA TFBR (Figure 2D)": I do not see/understand this point.

*Figure 2D shows higher densities of hypermethylated values in CEBPA’s TFBS distributions that do not appear for TFBRs. This indicates that a subpopulation of CEBPA TFBS have CpGs that are hypermethylated and this differs from the remaining TF’s distributions. To better elucidate this point, we generated a new figure panel named Supplementary Figure S1D where we show that the fraction of motifs with methylation above 75% exceeds the fraction of TFBRs with methylation above 75% only for CEBPA.*

Figure 2D line for dog is missing in the CEBPA TFBSs panel.

*We thank the reviewer for spotting this. Dog’s CEBPA line was indeed missing and a new Figure 2D has been generated.*

Figure 2D, is this specific to CEBPA? ONECUT and FOXA1 (Fig S1B) seem to have the same trend.

*It is indeed a general trend that the individual CpGs within the TFBRs and TFBSs have intermediate methylation levels but we selected CEBPA as a representative of this pattern. However, CEBPA is the only TF showing higher density of hypermethylation values within TFBSs as opposed to TFBRs.*

Page 5, should the title of the section not be "TF binding regions have LOW AND intermediate CpG methylation".

*The section title has been amended.*

Page 7, how does the title of the section differ from the title of the previous section?

*In the previous paragraph we meant to emphasise that TFs bind at DNA sequences with different methylation patterns which we were able to characterise, while in the next section we meant to highlight that these alternative methylation patterns correspond to different functional genomic contexts. We amended the paragraph titles to better reflect this.*

Figure 3A and S2A, B: Plots should all have the same scale for easier comparisons.

*We thank the reviewer for this suggestion, Figure 3A and S2A,B now all have the same scale.*

FigureS2A: For CEBPA in macaque, why is there no enrichment in CpG like for others? What is the quality of the ChIP-seq?

*As we mentioned in an earlier response to reviewer 1 and now explicitly address in the Result text, macaque’s CEBPA ChIP-seq data are indeed noisier than other TFs. However, the same trends are still present, and no specific result rests on the macaque ChIP-seq alonoe.*

Page 7 "This shows that TFBRs are characterized by key CpGs in close proximity of the binding site which are predominantly unmethylated when the protein is bound.": What are the "key" CpGs?

*As the CpG frequency profiles show increased CpG frequency in the immediate surrounding of the binding site despite the absence of prominent CpGs within the motif logos, we conclude that these CpGs must be important for the successful binding of the TF to their target sequence (hence, “key”). However, we agree that these “key” CpGs have not been further characterised so we removed the word “key” from the text.*

Figure 3 and S4 do not have the same color scale, legend and order of features, please correct.

*Figure 3 and S4 have been homogenized accordingly. Thank you.*

Figure 3D and S5: Genomic distances are usually represented in log10 scale (add pseudocount of 1 to see values at 0) with usually a bimodal distribution of the distance to TSS appearing below and above 2Kb. Can you try this scale?

*We thank the reviewer for this suggestion. Figure 3D is now in log10 scale. Indeed, the multimodal distribution appears, but it differs between TFBRs with distinct methylation profiles. We can now show more clearly that TFBRs in the flat clusters - which overlap active promoter regions - are more frequently distributed at shorter distances to TSSs. TFBRs in the remaining clusters have a mode at longer distances.*

What could explain the strong mirror effect of left/right clusters? There must be something that positions the regions. Could it be genes or other TF binding sites?

*We agree with the review that the strong mirror effect is an interesting property of these clusters. We further explored whether it can be explained with the directionality of transcription by overlapping the TFBRs with annotated TSSs and examining their direction of transcription. In Supplementary Figure S5A we now show that TFBRs with left profiles are more frequently located overlapping TSSs on the forward strand, while TFBRs with right profiles overlap those on the reverse strand, indicating that the lowly methylated regions of the profiles correspond to the first exon which is typically unmethylated.*

Page 10: "orthologous regions that do not bind a TF" should be "that are not bound by a TF"

*Thank you. It has been corrected accordingly.*

Figure 4B: Legend says methylation of "bound regions" or "orthologous unbound" but should it be "orthologous bound" like in the figure?

*Thank you. It has been corrected accordingly.*

Page 12 last paragraph and page 13 first paragraph: Hypothesis might not hold if analyses were performed on TF which motifs do contain CpGs. The TFs studied might not have CpGs but others binding to the same regions might. TF binding is accepted as a mechanism to initiate and maintain regions unmethylated.

*We agree with this comment and have now clarified in the text that the hypothesis might only hold for the TFs included in our study, but alternative mechanisms for TF with a direct interaction with methylated CpGs might exist.*

Do other TFs that also have CpGs in their motif have similar patterns than CTCF or is this really CTCF specific?

*We agree with this insightful comment and suggest in the main text that the analyses of different TFs with specificity rules more similar to CTCF might clarify whether the patterns that we see are ultimately CTCF specific.*

Your analysis of CTCF binding sites in different species could be compared to a previous one in different cell types: Role of DNA Methylation in Modulating Transcription Factor Occupancy. Maurano MT, et al. Cell Rep. 2015. PMID: 26257180

*We thank the reviewer for pointing out a relevant publication, and have used it to expand on the relevance of CTCF’s context effects and conservation in the Discussion. We use it to draw parallels between alterations of the DNA methylation landscape between different cell types and between species.*

Do direct binding sites (peaks that have the corresponding motif) have similar methylation patterns as indirect sites (peaks that to not have a motif)?

*Thank you for this interesting question. As shown below, there is not a striking difference between TFBRs with a discoverable motif within its region (“+ motif”) and TFBRs without (“- motif”). Both average methylation distributions (here in log scale) are bimodal, however, TFBRs without an annotated motif have higher densities both of unmethylated values and intermediately methylated values. We believe that these differences are merely due to the fact TFBRs with annotated motifs are a minority of all the TFBRs as our de novo motif identification strategy was indeed relatively stringent.*

In the introduction you mentioned that "20-40% of TF binding loss during evolution was not explained by mutations". Then, what is the part explained by DNA methylation? You could take this information into account in your analysis and separate loss and gains according to if they can be explained by mutations or not and then look at methylation levels in those two groups.

*This is an extremely important point that we have considered along the way, but did not pursue due to insufficient sample size to make statistically robust conclusions about the effect of DNA methylation on TF binding loss. For this analysis, we would need CpGs at the binding site of TFBRs which were evolutionarily lost. Firstly, less than 10% of TF binding motifs contain at least one CpG which could be methylated (Fig 2A). This set is even smaller if we consider that the motif would need to be completely conserved, including the CpG site, to unequivocally assess only the effect of methylation on binding divergence. Finally, there is an inherent bias in the distribution of CpG-motifs within different genomic contexts. A larger fraction is located within CpG denser regions - like promoters - rather than other CpG poorer contexts (figure below). Promoters are the most evolutionarily conserved genomic contexts, are consistently unmethylated and functionally conserved.*

**Third round of review**

**Reviewer 2**

I have read with attention your comments. Although there might be CpGs in some positions of the motifs (at position 6 instead of 7 for CEBPA). The fraction of TF motif occurrences containing motifs (Figure 2A) remains very limited from 3 to 18% and up to 30% for CTCF.
Although you state that the surrounding regions do contain methylated CpG, most of the genome does and it was previously shown that it is the methylation within the TF motif specifically that affect binding (PMID: 34234345, PMID: 26675734). Some other TFs that have binding sites in the surrounding regions might be affected and affect the cooperativity necessary for binding but this is not explored in your study.
I acknowledge that you do observe anticorrelation across species between TF binding and DNA methylation but this is not sufficient to state (in your rebuttal) that TF binding is affected by DNA methylation as it could as well be that it is DNA methylation that is affected by TF binding. This might actually be the case for FOXA1, which is a known pioneer TF. Therefore, a possible explanation for the changes across species could also be that loss or gain of TF binding leads to gain or loss of DNA methylation. This could also well be due to loss or gain of cooperative TF binding with DNA methylation changes being only a consequence.
In summary, the anticorrelation observed between TF binding and DNA methylation across species is interested but expected and does not imply that DNA methylation changes lead to TF binding changes especially since the TFs studied do not have prominent CpG directly in their motifs. This is why I would reject this manuscript as not enough priority for Genome Biology.
